# Protocol variations in run-on transcription dataset preparation produce detectable signatures in sequencing libraries

**DOI:** 10.1186/s12864-022-08352-8

**Published:** 2022-03-07

**Authors:** Samuel Hunter, Rutendo F. Sigauke, Jacob T. Stanley, Mary A. Allen, Robin D. Dowell

**Affiliations:** 1 BioFrontiers Institute, University of Colorado, Boulder, 80309 USA; 2 Computational Bioscience Program, Anschutz Medical Campus, University of Colorado, Aurora, 80045 USA; 3 Molecular, Cellular, and Developmental Biology, University of Colorado Boulder, Boulder, 80301 USA; 4 Department of Computer Science, University of Colorado, Boulder, 80309 USA

**Keywords:** Run-on sequencing, PRO-seq, GRO-seq, Library preparation

## Abstract

**Background:**

A variety of protocols exist for producing whole genome run-on transcription datasets. However, little is known about how differences between these protocols affect the signal within the resulting libraries.

**Results:**

Using run-on transcription datasets generated from the same biological system, we show that a variety of GRO- and PRO-seq preparation methods leave identifiable signatures within each library. Specifically we show that the library preparation method results in differences in quality control metrics, as well as differences in the signal distribution at the 5 ^′^ end of transcribed regions. These shifts lead to disparities in eRNA identification, but do not impact analyses aimed at inferring the key regulators involved in changes to transcription.

**Conclusions:**

Run-on sequencing protocol variations result in technical signatures that can be used to identify both the enrichment and library preparation method of a particular data set. These technical signatures are batch effects that limit detailed comparisons of pausing ratios and eRNAs identified across protocols. However, these batch effects have only limited impact on our ability to infer which regulators underlie the observed transcriptional changes.

**Supplementary Information:**

The online version contains supplementary material available at (10.1186/s12864-022-08352-8).

## Background

The transcriptome dictates much of a cell’s identity and behavior. As such, tracking how transcription patterns change in response to a biological perturbation is a popular approach to understanding molecular regulatory mechanisms. In particular, newly transcribed RNAs provide a readout on the activity and regulation of cellular RNA polymerases. Capturing and mapping these “nascent” transcripts provides a single base-pair resolution readout of the positions of all cellular RNA polymerases throughout the genome [1–3]. Changes in RNA polymerase behavior are associated with transcription factor activity [4–6], with a large portion of transcriptional changes occurring within enhancer regions. These enhancer RNAs (eRNAs) are unstable and thus not generally recovered by steady-state assays such as RNA-seq, which sample predominantly from the pool of stable transcripts such as mRNAs [7].

**Fig. 1:**
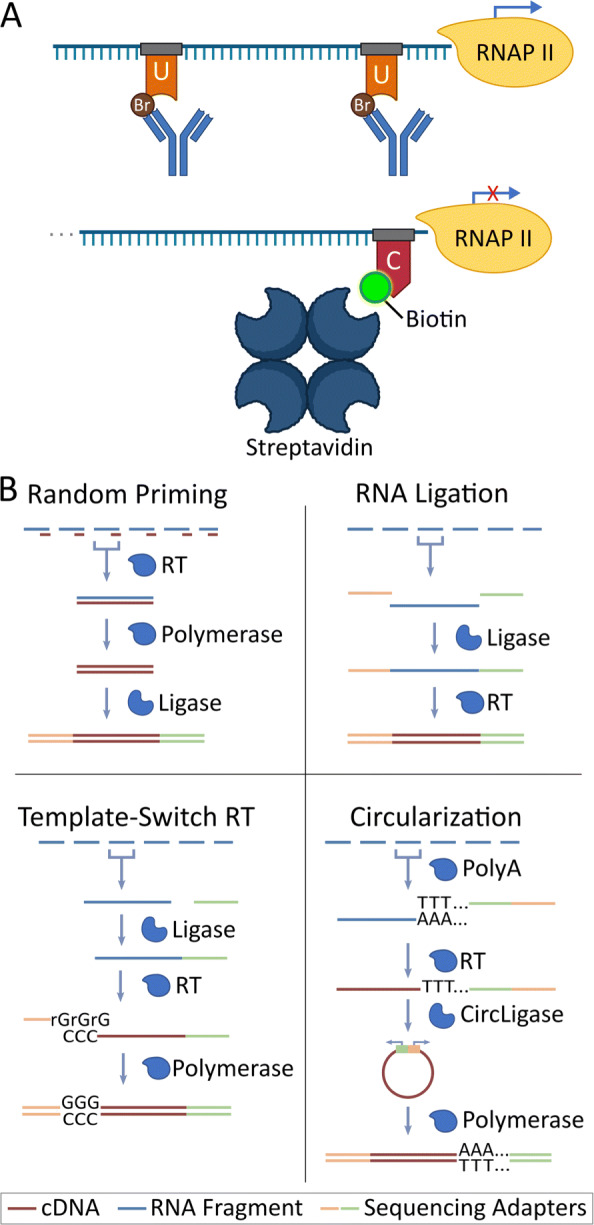
Summary of Run-On Sequencing (RO-seq) data sets. **A** Summary diagram indicating enrichment steps for Global Run-On (GRO-seq, top) and Precision Run-On (PRO-seq, bottom) reactions. **B** Summary diagram for library preparation reactions. Blue bars: RNA; brown bars: cDNA; yellow/green bars: sequencing adapters. Library preparation enzymes are labeled and represented by blue shapes at each step

To capture all RNAs arising from cellular RNA polymerases, several run-on transcription capture protocols, such as global run-on sequencing (GRO-seq) and precision run-on sequencing (PRO-seq), have been developed [1, 3, 8–10]. These protocols, collectively known as RO-seq, follow roughly a two step process: first, the run-on RNA signal must be enriched above the background total RNA; second, the captured RNA is then converted into a sequencing-ready cDNA library [1]. For the first step, run-on protocols share the same basic strategy, namely they use an enrichable nucleotide as a handle for distinguishing nascent RNA from previously produced RNA (Fig. 1A). Subsequently, sequencing adapters are added and the sample is reverse transcribed and amplified in preparation for sequencing. As these steps are somewhat modular, the process of enrichment is often interleaved with the various steps necessary for library preparation (Fig. 1B).

Similar to distinct RNA-seq library preparation methods, processing RNA through different RO-seq protocols is thought to leave technical artifacts within the library [11–13]; however, the extent to which these artifacts influence the resulting analysis has not been thoroughly explored. In this study, we sought to identify specific signatures and biases inherent to the protocol (enrichment strategy) and library preparation methods typically employed in RO-seq methods. For this comparison, we generated data from HCT116 cells treated for 1 hour with the p53 activator Nutlin-3a or a DMSO control, a well studied perturbation [4, 14]. Using these matched datasets, we find specific and reproducible biases in each respective dataset that influence both the quality metrics and 5 ^′^ distribution of reads. However, we find that these protocol and library specific effects do not strongly impact the inference of which transcription factor is driving the observed perturbation induced changes in transcription. These protocol-specific signals could enable an agnostic detection program to identify the protocols used; such programs could then be utilized to increase the validity of online sequence databases.

## Results

### Quality metrics are influenced by RO-seq transcription capture protocols

The ultimate goal of run-on protocols is to produce a dataset that accurately reflects the distribution of actively transcribing RNA polymerase [1, 15] genome wide. However, success in this endeavor depends greatly on the sequencing depth, library complexity, quality of enrichment, and transcription strength of the cell line [16]. To control for cell line differences, we generated run-on libraries from HCT116 cells using a previously employed perturbation strategy [4, 14]. Namely, we used global run-on (GRO) sequencing [1] with a Br-tagged UTP, and precision run-on (PRO) sequencing [2] with a Biotin to mark CTP [3] (Fig. 1A) as enrichment protocols. We then combined these enrichment protocols with one of four library preparation techniques: RNA adapter ligation (LIG) [1], Circularization (CIRC) [8], Random Priming (RPR) [17], or Template-Switching Reverse Transcription (TSRT) [9] (Fig. 1B) after either 1 hr DMSO control or 1 hr treatment with Nutlin-3a. Nutlin-3a is a molecule which interrupts p53 inhibition and leads to rapid transcription of downstream p53 targets (see Materials and methods). Samples were subsequently sequenced on an Illumina NextSeq 500 platform (RTA version: 2.4.11, Instrument ID: NB501447) using a single end strategy (37, 50 or 75 bp lengths) to variable depths (summarized in Supplemental Table 1, see Materials and methods).

The first noticeable differences between any two datasets (even with the same protocol and library preparation) are depth of sequencing and complexity of the library. The depth of our samples range from 20 million to 170 million reads. We correct for the disparity in sequencing depth by combining the technical replicates of low-depth samples, and by subsampling deeply sequenced samples. As such, subsequent comparisons were performed at equivalent depth (with an average of approximately 70 million reads for GRO-LIG, PRO-LIG, and GRO-CIRC library comparisons, and a minimum of 20 million reads for GRO-RPR library comparisons).

In contrast, library complexity reflects data quality and cannot be corrected for computationally and ideally would be similar between library preparations before comparison. We use two metrics to assess complexity, the number of unique reads relative to the depth of the sample and the number of unique bases covered within the genome (Supplemental Table 1). While most of our libraries were comparably complex, we found that our libraries generated with a random-priming library kit were generally of lower complexity. The random-priming strategy is rarely used and thus, it is unclear whether the tendency of reduced complexity is a consequence of the library preparation method or a fault of our handling. However, public random primed datasets [18] exhibited similar 5 ^′^ read distributions to our datasets in spite of the differences in library complexity (Supplemental Figs. 1, 2, 3, Supplemental Table 1); therefore, we chose to include these libraries in our initial analyses to showcase possible technical signatures and potential biases, but refrained from using GRO-RPR libraries in further comparative analyses.

**Fig. 2:**
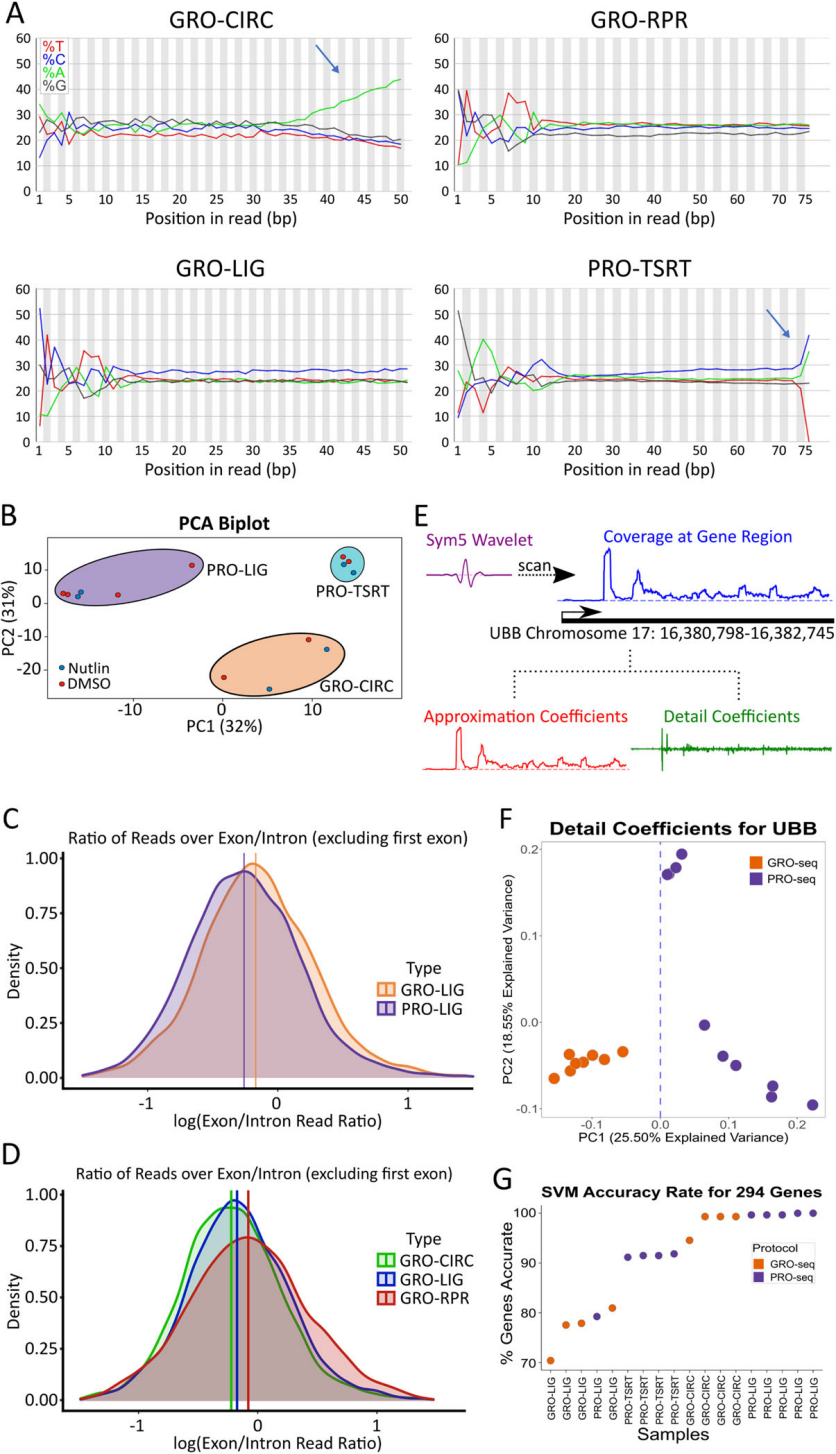
Quality Control metrics for varying library preparation and enrichment techniques. **A** Nucleotide distribution of DMSO samples are plotted indicating the percent nucleotide representation (y-axis) versus the position within each read (x-axis). Library specific signatures are identifiable in CIRC and TSRT libraries (blue arrows). **B** Principal-Component Analysis of assorted library preparation and enrichment methods. Each library was prepped using HCT116 cells treated with either DMSO or Nutlin-3a for 1 hour. Log-normalized density plots of exon/intron ratios for each gene for each **C** enrichment method and **D** library preparation method (GRO-seq samples shown), (GRO-LIG vs PRO-LIG: p <.001; GRO-CIRC vs GRO-LIG: p <.05; GRO-CIRC vs GRO-RPR: p <.001; GRO-RPR vs GRO-LIG : p <.001, K-S Test, n=1795). Mean indicated by vertical line for each respective distribution. **E** Schematic showing the wavelet transformation approach at the UBB locus. **F** Detail coefficients at UBB locus separates PRO and GRO libraries on PC1 (Low-biotin PRO-seq samples omitted, see Supplemental Table 1). **G** SVM classifier results for each tested library

Notably, some library preparations result in clearly distinguishable sequence signatures within the acquired reads. In circularization (CIRC) libraries, regardless of the enrichment protocol, RNA is polyadenylated before reverse transcription, and the resulting cDNA is subsequently circularized via the enzyme circLigase [8]. As such, it is common to see many reads with long poly(A) tails before trimming (Fig. 2A). Additionally, the TSRT library preparation adds several C nucleotides to the end of each read [9]. Upon sequencing and adapter trimming, many read inserts showed an increased incidence of C nucleotides near the end of the read (Fig. 2A). In our samples, these sequence signals can effectively distinguish CIRC and TSRT libraries from the other library preparation methods. In contrast, LIG and RPR libraries show similar nucleotide composition across the reads. Likewise, GRO and PRO datasets constructed with matched library preparation methods are not distinguishable from sequence content signatures alone.

However, principal component analysis (PCA) of the read counts over all genes tightly clusters based on library preparation and enrichment protocol, suggesting there are additional protocol-distinguishing features not evident in the average nucleotide composition of the dataset (Fig. 2B). Therefore, we next sought to identify whether enrichment quality metrics could be used to distinguish between the protocols. Quality control pipelines offer a way of quantifying steady-state RNA contamination by calculating the ratio of reads over exons and introns for each gene. While the specific value expected for this ratio depends on how reads are counted, a comparatively lower exon-intron ratio is indicative of less mRNA contamination [19]. But is this exon-intron ratio influenced by the choice of protocol? To answer this, we calculated log-normalized exon-intron ratios for every gene in each HCT116 control (DMSO) library. On average, PRO libraries showed a slightly lower amount of mRNA contamination across all genes relative to GRO libraries, consistent with the relative strength of the two enrichment strategies (Fig. 2C). Additionally, both CIRC and LIG libraries showed lower mRNA contamination relative to RPR libraries (Fig. 2D).

Sequence composition (Fig. 2A) can be utilized to identify CIRC and TSRT library preparation protocols with high confidence, while LIG and RPR libraries were more similar in sequence composition, albeit with some differences in complexity and quality metrics (Fig. 2D, Supplemental Table 1). However, the differences between the enrichment protocols (GRO vs PRO) is less readily apparent from sequence composition or quality metrics alone (Fig. 2A,C, Supplemental Table 1). Yet, we wondered whether systematic signals exist within the data that could distinguish between the protocols. To this end, we applied a discrete wavelet transform (DWT) approach to the normalized coverage of each library (Fig. 2E). The DWT decomposes the signal in a region into low frequency signals (approximation coefficients) that capture consistent RNA polymerase signatures and high frequency signals (detail coefficients) that contain noise. The noise component captures both random noise and systematic noise. Because protocol specific signatures are a systematic source of noise, we reasoned that the high frequency signals may be able to distinguish between the protocols.

To test this hypothesis, we sought to evaluate the DWT on a set of genes where RNA polymerase signatures are the least influenced by library depth or complexity. Thus we identified a set of 294 highly transcribed genes that also had a low coefficient of variation across our datasets. Using the PyWavelets package in python, a symlet wavelet was scanned over the normalized coverage of each gene, effectively decomposing the signal into the two components (see Materials and methods) (Fig. 2E) [20, 21]. Subsequently, we used principal component analysis (PCA) to cluster the detail coefficients. Overall, 117 genes (39.8%) separated the protocols (GRO vs PRO) directly on the first principle component whereas an additional 162 (55.1%) genes separated the protocols on a different plane within the PC1 and PC2 space (Fig. 2F, Supplemental Fig. 4, 5). These results suggested that the data sets contain a readily identifiable protocol signature. To confirm, we built a simple support vector machine classifier to determine whether the principle components of the wavelet analysis could be used to identify the protocol directly from the data (see Materials and methods) (Supplemental Fig. 6). Using leave-one-out cross validation at the individual gene level, the classifier correctly identified the protocol >70% of the time (Fig. 2G, Supplemental Fig. 7). Furthermore, applying a simple majority rules voting scheme to the classifier results identified the protocol every time (100%), further confirming that each data set contains identifiable protocol specific signatures.

### Enrichment and library preparation methods significantly shift 5^′^ distribution

**Fig. 3:**
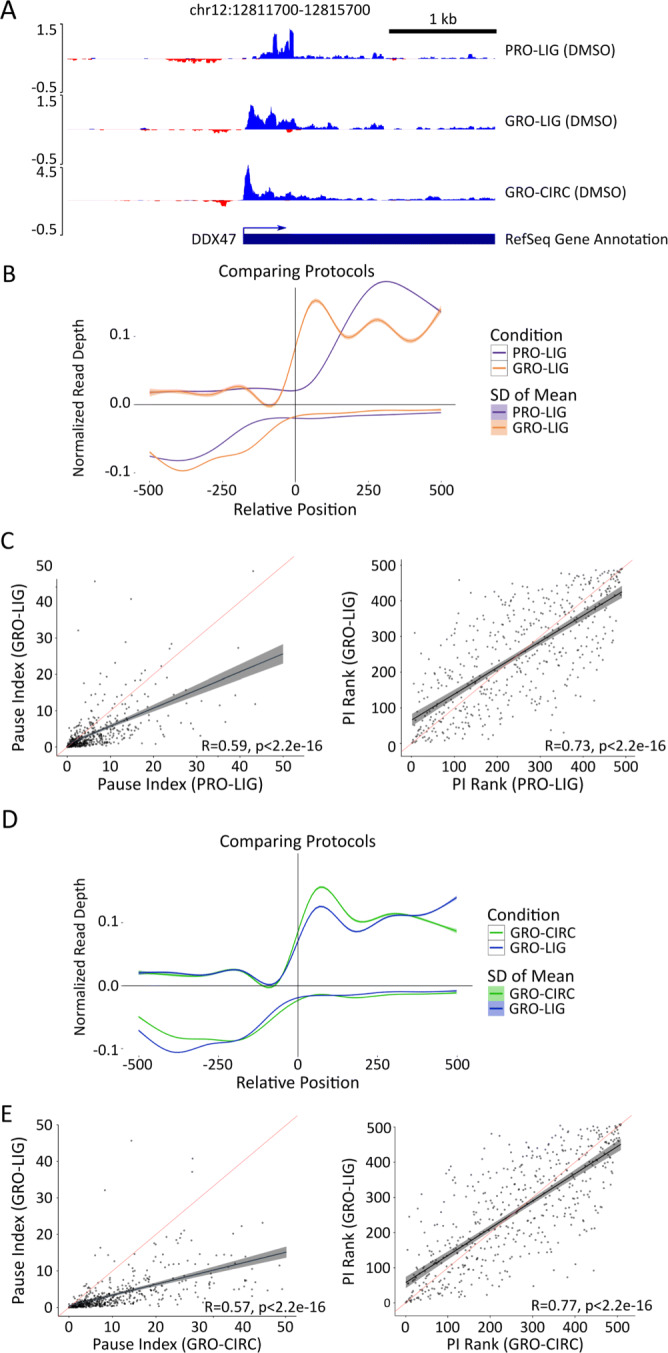
Analysis of gene transcription start sites among different protocols and library preparations. **A** Genome viewer screenshot of 5^′^ end distribution among various library preparation and enrichment methods. Negative read depth represents reads found on the minus strand. **B** Metagenes constructed from GRO-seq (orange) and PRO-seq (blue) libraries (Ligation based library preparation, HCT116, DMSO 1hr). Genes shorter than 2000 bp were removed, genes with significant signal 2 kb upstream (>1% of upstream bases covered), and genes with low coverage (TPM <.01) were removed (n=2527). Vertical line indicates TSS annotated in RefSeq database. Distance from TSS is in bp, read depth was normalized by counts-per-million (CPM). **C** Pausing index calculations for top 500 most transcribed genes in GRO-seq and PRO-seq libraries, presented with Pearson (left) and Spearman (right) correlations (red line: y=x, black line: best fit). Pausing region is defined as -50 bp to 250 bp from annotated TSS (See Materials and methods). **D** Metagenes constructed from GRO-seq Ligation (blue), and Circularization-based (green) libraries (HCT116, DMSO 1 hr). Genes shorter than 2000 bp, genes with significant signal 1 kb upstream (>1% of upstream bases covered), and genes with low coverage (TPM <.01) were removed (n=2527). Vertical line indicates TSS annotated in RefSeq database. Distance from TSS is in bp, read depth was normalized by counts-per-million (CPM). **E** Pausing index calculations for Circularization and Ligation based libraries (GRO-seq, HCT116, DMSO 1 hr), graphed as in (**C**)

To better understand the protocol specific signatures within the data sets, we next examined annotated, protein-coding genes for systematic differences in their read distributions. At protein-coding genes, the behavior of RNA polymerase II is well characterized [22] which leads to repeatable patterns of read distribution throughout the gene (Fig. 3A). Therefore, we sought to determine whether the protocol (GRO vs PRO) led to systematic differences in the detected 5^′^ initiation region or the elongation region. Counts across gene body regions suggested that elongation regions correlated well between protocol and library preparation differences (Supplemental Fig. 8, see also Materials and methods); therefore, we subsequently focused our attention on the 5^′^ regions of genes.

To assess the differences in the 5^′^ distribution across protocols, we examined the read distribution of GRO and PRO libraries prepped from DMSO-treated HCT116 cells, with an otherwise similar library preparation protocol (LIG). Metagenes revealed a shift in coverage near many transcription start sites (TSS) in PRO libraries that is not present in GRO libraries (Fig. 3B, Supplemental Fig. 9). GRO and PRO libraries differ in the nucleotide analog used to enrich for nascent RNA. In GRO-seq, bromouridine-triphosphate is used to mark newly transcribing RNAs which can then be detected by anti-BrdU antibodies. In contrast, PRO-seq uses Biotin-NTPs which also terminate transcription upon their incorporation into the nascent RNA. Streptavidin then efficiently isolates newly transcribed RNAs. The original PRO-seq strategy marked all four nucleotides to maximize precision [1], but for cost efficiency, subsequent efforts only marked a single nucleotide [3]. Notably, both the efficiency of pull down and the termination of transcription results in PRO-seq giving a more precise readout on the position of RNA polymerases relative to GRO-seq [2]. However, at the 5^′^ end this precision also results in short unmappable reads, leading to gaps in coverage near the TSSs [3]. In an attempt to mitigate these 5^′^ read coverage gaps, subsequent variations in the PRO-seq protocol include a ratio of Biotin-NTP/NTP to the run-on mixture [3].

We theorized that the shift in the 5^′^ region observed in our PRO libraries arose from early incorporation of Biotin-NTP near the TSS which leads to short, truncated reads that are not well mapped. As such, we reasoned that generating new libraries with a different ratio of Biotin-NTP/NTP in the initial run-on mixture would result in more reads captured around the 5^′^ end (Supplemental Fig. 10). Metagenes indeed show a smaller shift with lowered Biotin-NTP concentration, although GRO-LIG libraries continued to show more signal in these regions than any PRO library.

To ensure that our findings generalize to other data sets, we next examined publicly available datasets. While these data sets likely have larger batch effects arising from their preparation in distinct laboratories and cell types, we reasoned that the overall trend in 5^′^ end patterns should still be noticeable, albeit subject to more variance. GRO and PRO libraries obtained from other labs showed that the peak of PRO-seq libraries was noticeably further downstream than their GRO-seq counterparts; however, this comparison (using a consistent mapping and analysis strategy, see Materials and methods) uncovered a broad range of peak positions (from +40 bps to +250 bps) with seemingly no linear relationship between the Biotin-NTP/NTP ratio and peak position (Fig. 3B, Supplemental Figs. 10, 11, 12).

Therefore, we reasoned that there must be further underlying protocol influences on the 5^′^ read distribution. Differences in size selection, read fragmentation, and gene filtering criteria were all hypothesized to influence the distribution. To evaluate these criteria, we took an in silico approach and simulated reads arising near a TSS from each protocol configuration (see Materials and methods). Briefly, positions of potential polymerase occupancy were sampled from a simulated gene, including both initiation and elongation regions. For each polymerase position, we extended the hypothetical RNA based on the gene template downstream of the polymerase position, with the designated probability of incorporating a Biotin-NTP and halting extension. The subsequent read was then filtered by size selection and plotted to generate simulated metagene traces (Supplemental Fig. 13). Using these simulations, we found that the 5^′^ peak position was influenced by both the Biotin-NTP run-on ratio and the size selection criteria.

To validate our in silico findings, we returned to the data and examined the distribution of short reads (less than 30 bps) relative to transcription start sites. We reasoned that short fragments would consist of a combination of TSS associated fragments truncated by Biotin-NTP incorporation and small fragments arising from sample handling, which should be randomly distributed throughout the genome. Hence the ratio of short reads near TSS relative to all short reads should be indicative of the ratio of labeled and unlabeled NTPs used in the run-on reaction. Indeed, the short read ratio does shift along the Biotin-NTP ratio, but not as a monotonically increasing function (Supplemental Fig. 14). Consistent with our simulations, intermediate Biotin-NTP/NTP ratios returned the highest fraction of mappable TSS associated short reads. Our results indicate that several library preparation elements, such as size selection, Biotin-NTP run-on ratios, and mappability strongly influence the 5^′^ distribution. Importantly, this work also suggests that the ideal run-on scenario is a balance between producing reads that are long enough to escape size selection and map effectively, yet remain short enough to accurately report on the position of RNA polymerase.

We next reasoned that the observed differences in the detected 5^′^ read distribution at genes would commensurately affect the pausing index (PI), measured as the ratio of reads in the initiation region relative to the gene body [23]. We defined the initiation region as 50 bp upstream from the annotated TSS to 250 bp downstream of the TSS; gene body regions were defined as 251 bp downstream of the TSS to the annotated cleavage site. Using these regions, we calculated the PI for the longest isoform of each gene in both libraries. Consistent with our findings above, PI for individual genes were reasonably consistent across replicates (Supplemental Fig. 15) but showed significant disparities between GRO and PRO libraries (Fig. 3C, R = 0.59, p < 2.2e-16). Spearman rank correlations for PI in both libraries were marginally higher (R = 0.73, p < 2.2e-16). These overall trends were also observed within PI distributions when we extended this analysis to publicly available data (Supplemental Fig. 12).While the PI is known to depend on the method used to define the paused region [15, 24], we found that the trends across protocols remained consistent even with different pause windows and read counting software (Supplemental Fig. 16).

Next, we evaluated the effects of library preparation on the 5^′^ end. To accomplish this, we constructed metagene summaries of our GRO-CIRC and GRO-LIG libraries (Fig 3D). CIRC and LIG libraries showed a similar distribution near the 5^′^ end. When GRO-RPR libraries were compared to GRO-LIG and GRO-CIRC libraries, however, GRO-RPR libraries show a shift in coverage that leaves a significant gap near the annotated start site (Supplemental Fig. 2). While it is unknown what leads to this shift, we theorize that random priming has a length bias that is a contributing factor (i.e. the longer a RNA is the more likely a primer is to anneal to it).

Additionally, we found that the pause ratio is sensitive to which method is used to prepare the RNA. We compared pause index calculations for GRO-CIRC and GRO-LIG libraries. We found that, for each gene, pause indices tended to be larger for GRO-CIRC libraries compared to GRO-LIG libraries (Fig. 3E, R = 0.57, p < 2.2e-16). To assay whether this shift was systematic, we also computed the Spearman rank-correlation for these indices. Rank correlation between GRO-LIG and GRO-CIRC libraries was stronger than Pearson correlation; however, there were still many genes that showed disparate rankings across our datasets (Fig. 3E, R = 0.77, p < 2.2e-16).

### Changing library enrichment methods shifts intergenic read distributions and active enhancer detection

**Fig. 4:**
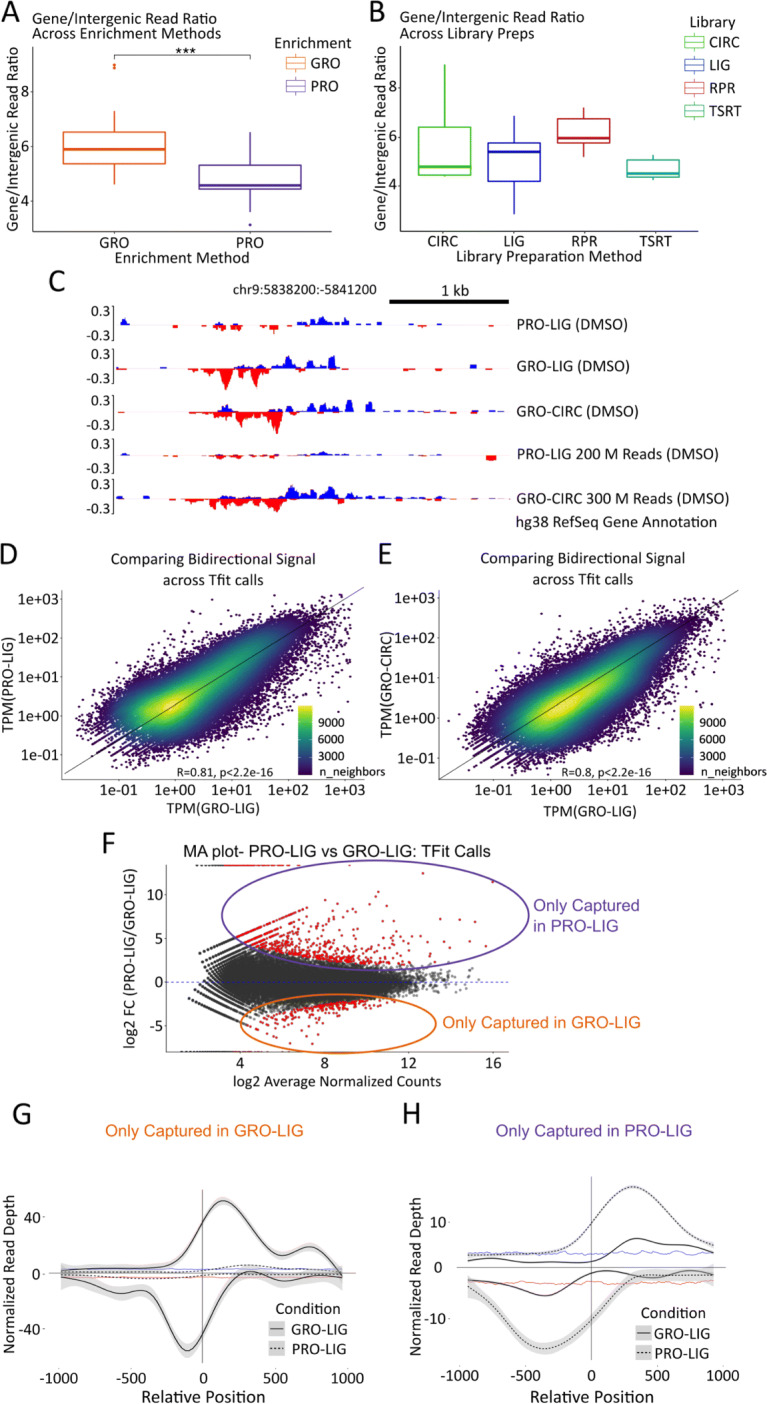
Analysis of enhancer elements in multiple datasets. **A**, **B** Number of reads counted over RefSeq annotated gene regions divided by the number of reads counted over intergenic (unannotated) regions, for each dataset analyzed. The datasets represented here are all those listed in Supplemental Table 1, including public datasets. Datasets were first analyzed by enrichment method (GRO-seq (n=23) vs. PRO-seq (n=21), p <.01), then by library preparation method (LIG (n=17) vs CIRC (n=10) vs TSRT (n=10) vs RPR (n=7), p >.05). We note that the RPR boxplot includes 3 of our lower quality datasets; however, we chose to include them here owing to the scarcity of RPR datasets in the RO-seq database. These are otherwise excluded from further analysis. **C** Example section representing disparate representation of reads from our in-house datasets over an enhancer, even at high depths. **D**, **E** Scatterplots representing reads over Tfit (enhancer) calls (calls combined by MuMerge, counts normalized by TPM). **F** MA plot of calls found in (**D**). Red dots are significant (p <.05). **G**, **H** Metagenes of significant hits found in (**F**). Vertical line indicates the approximated center of the bidirectional transcripts as determined by Tfit. Distance from the center of the bidirectional is in bp, read depth was normalized by counts-per-million (CPM). **G** Calls that were differentially captured in GRO-LIG (n=1350). Background signal on the plus strand is indicated by the blue trendline, while background signal on the minus strand is indicated by the red trendline. **H** Calls that were differentially captured in PRO-LIG (n=3050), with the background signal depicted as in panel **G**

The bidirectional transcription typical of RNA polymerase initiation regions at the 5^′^ end of genes is also present at enhancers [25], albeit typically at much lower transcription levels. Therefore, we asked whether the patterns of enhancer transcription varied across protocols or library preparations. As a first pass inquiry that avoids reliance on enhancer annotations, we first compare the fraction of reads recovered from RefSeq annotated gene regions to reads recovered in intergenic regions for each data set. To ensure more statistical rigor, we included several publicly available datasets of different cell lines, along with six libraries we previously generated from MCF10A cells prepped with PRO-TSRT (See Supplemental Table 1). When comparing GRO and PRO libraries (irrespective of cell type or library preparation method), we found that GRO libraries showed significantly more reads over gene regions compared to PRO libraries (Fig. 4A, p <.01, See Materials and methods). Conversely, we found no significant differences when comparing library preparation methods (Fig. 4B).

The disparity in the gene-to-intergenic reads ratio in GRO and PRO libraries suggest their respective enrichment strategies may capture signal in unannotated regions at different rates. In particular, we were curious whether the capture of eRNAs would be affected by the choice of protocol. To investigate this possibility, we first examined annotated enhancers in the HCT116 cell line acquired from the FANTOM database (converted to hg38 coordinates using the online UCSC tool liftOver) [26]. The level of transcription between these enhancers was largely consistent between our datasets (Supplemental Fig. 17). However, FANTOM annotated enhancers represent the comparatively stable enhancer transcripts arising from Cap Analysis Gene Expression (CAGE) data [27].

Therefore, we next sought to identify enhancers directly from the data using their characteristic bidirectional transcription signal [28]. Two algorithms have been developed to identify transcribed regulatory elements based on their bidirectional signal, dREG [29] and Tfit [30]. We employed both methods to annotate sites of bidirectional transcription in our GRO-CIRC, GRO-LIG, and PRO-LIG libraries. Strikingly, the identified regions varied substantially across protocol and library preparation for both algorithms (Supplemental Fig. 18). We hypothesized that these differences may be exaggerated by the sequencing depth, as eRNAs are lowly transcribed and therefore these regions are only consistently detectable at high sequencing depth. To this end, we combined replicates for PRO-LIG libraries to an effective depth of approximately 200 million reads, and replicates of GRO-CIRC libraries to an effective depth of approximately 300 million reads. Transcribed regions identified in these combined libraries remained inconsistent; while many strong enhancers were called in both of these two deep data sets, other regions were exclusively found in only one (Fig. 4C, Supplemental Fig. 19).

This suggested the existence of transcribed regions whose signal is strongly dependent on the underlying experimental protocol. To confirm this possibility, we next sought to identify the set of transcribed regions with apparent differential transcription across protocols or library preparations. To compare enrichment protocols, we combined Tfit regions from PRO-LIG and GRO-LIG libraries (Fig. 4D, Supplemental Fig. 17, see Materials and methods), while library preparation methods were compared by combining Tfit regions from GRO-LIG and GRO-CIRC libraries (Fig. 4E). In every case, regions were combined using *muMerge* [6] and differential read signal was assessed with DESeq1 analysis (Fig. 4F). We then constructed metagenes from set of regions with differential signal (Fig. 4G, H, Supplemental Fig. 20) and observed strong bidirectional signal in only one of the two datasets, while the other dataset showed signal only slightly above background. Manual inspection confirmed that these transcribed regions were only effectively captured by one library, even at high depths (Fig. 4C).

### Biological response to p53 activation is preserved across run-on transcription capture protocols

The protocol-specific nature of both pausing ratios and eRNA recovery led to concerns about whether the choice of experimental preparation influences commonly conducted downstream analyses, such as identifying which genes respond to a perturbation [4] and which transcription factors drive those changes [5, 6, 31, 32]. As such, we used the competitive MDM2 inhibitor Nutlin-3a, which has a known, specific, robust transcription response in human cells induced by the subsequent activation of the transcription factor p53 [4, 14, 33].

**Fig. 5:**
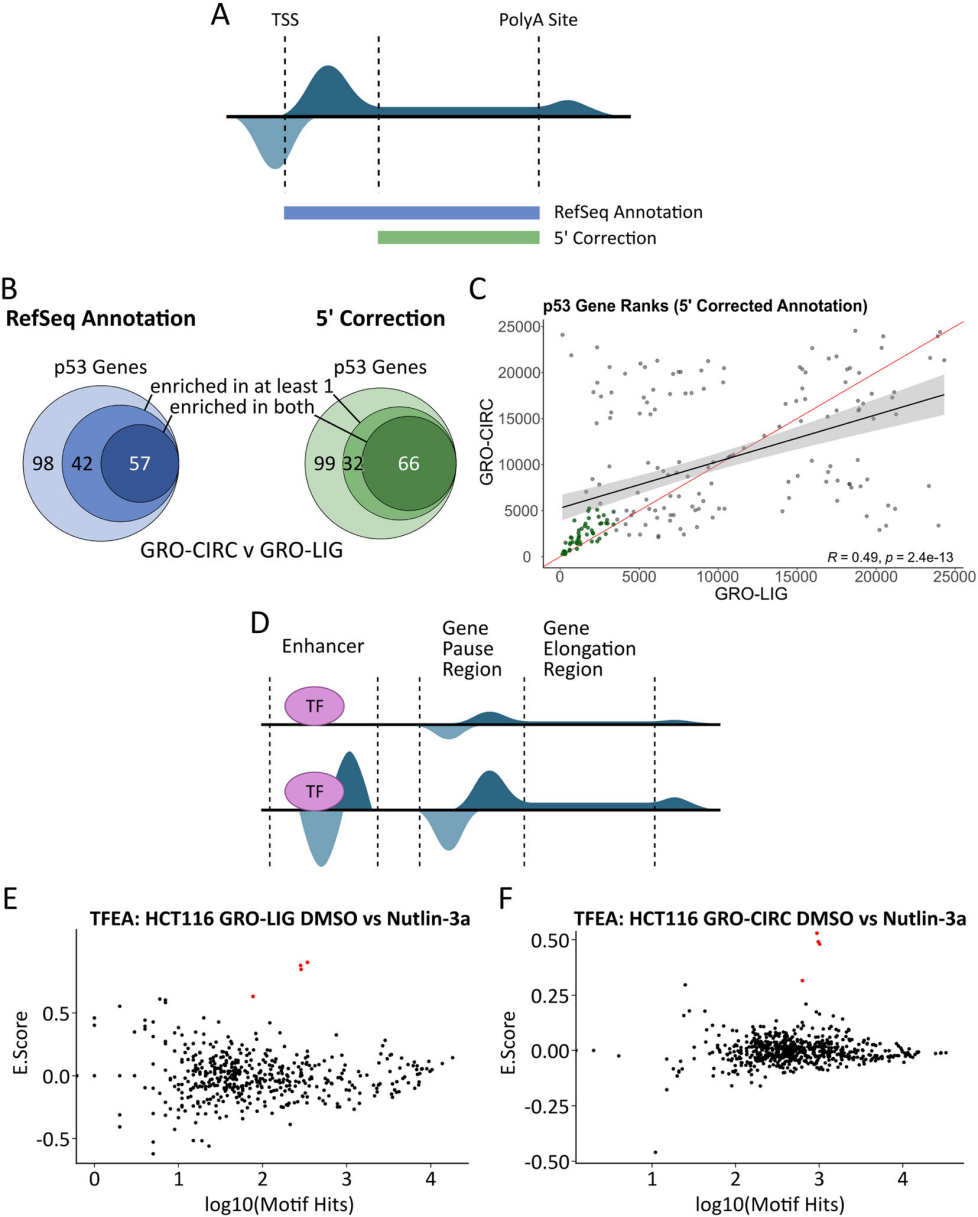
TFEA and DESeq2 analyses of library preparation methods. **A** Cartoon schematic demonstrating uncorrected (RefSeq Annotation) and 5^′^ corrected counting methods. **B** GSEA gene rank comparison of HALLMARK_P53 Gene set. Overlap is shown as genes that enrich in both datasets, genes that enrich in only one dataset, and genes that do not enrich in either dataset (Left: Uncorrected annotation, hypergeometric test *p*-value=4.32e-15; Right: Corrected annotation, hypergeometric test *p*-value=9.03e-22). **C** Scatterplot of comparative gene ranks for all p53 genes. Points in green indicate significant enrichment, as in (**B**). (Red line: y=x trendline, black line:line of best fit). **D** Representation of nascent transcription data set. Bidirectional transcripts occur at active enhancer sites and gene start sites. Enhancer transcription co-occurs with upregulated gene transcription, indicating transcription factor activation. **E** TFEA results for GRO-LIG (Left) and GRO-CIRC (Right). p53 family (p53, p63, p73) highlighted by red dots

First, we sought to determine the reproducibility of detecting differential gene transcription within our libraries. The precise identity of which genes respond to 1 hour of p53 activation is expected to vary across protocols and library preparations – as similar batch effects have been observed for RNA-seq libraries [34]. Thus, we focused specifically on whether the core p53 response program, i.e. the known targets of p53, was captured efficiently in each dataset. To this end we utilize the Gene Set Enrichment Analysis (GSEA) - Preranked [35, 36] tool on ranked, signed *p*-values obtained from DESeq2 [37] (See Materials and methods). Additionally, we expected that a substantial amount of variation between two libraries generated from different protocols would arise from the gene initiation region (Fig. 3). To confirm this, we subsequently examined two distinct methods of calculating differential gene transcription: the commonly used elongation-region-only approach and the full annotated gene region (Fig. 5A). Across all libraries and counting methods, the p53 pathway was the top hit in the GSEA-Preranked module (FDR q-val < 0.001, Fig. 5B, Supplemental Fig. 21), suggesting that each protocol, library preparation and counting method was capable of detecting the underlying biological perturbation in spite of technical signals introduced by protocol differences.

Next, we compared the correlation of the ranks of the genes in the Hallmark p53 pathway used by GSEA. We found that the majority of enriched genes were common between each of the libraries (58.3% in GRO-LIG vs GRO-CIRC, 57.1% in GRO-LIG vs PRO-LIG) (Fig. 22B,C, Supplemental Fig. 22). However, there remained several genes that were only enriched in one of libraries. When only the elongation region was considered, the overlap improved (68.3% in GRO-LIG vs GRO-CIRC, 58.9% in GRO-LIG vs PRO-LIG), consistent with the 5^′^ initiation regions being the most variable portion of the gene between protocols. These results add further support to the most common method of assessing differential transcription from run-on sequencing protocols, namely excluding the 5^′^ initiation regions [38–41].

The second typical use of run-on sequencing data is to infer which regulators are driving observed patterns of differential transcription [5, 25, 29]. Alterations in transcription factor activity can be detected by changes in the locations and levels of sites of bidirectional transcription [5, 6] (Fig. 5D), the majority of which reside at enhancers [28]. Therefore we next sought to determine whether the alterations observed in eRNA detection (Fig. 4) impacted TF activity inference [6].

To this end, we used the Transcription Factor Enrichment Analysis (TFEA) tool to evaluate which transcription factor motifs are enriched at transcription initiation sites with altered transcription levels in response to Nutlin-3a [6]. In all cases, TFEA correctly identifies the p53 family (TP53, TP63, and TP73) as significantly upregulated, independent of the protocol and library prep used to generate the dataset (Fig. 5E and F, Supplemental Fig. 23). Upon closer inspection, 94.59% of p53-responsive enhancers responded similarly across protocols, but 5.41% of p53-responsive enhancers were unique to a particular protocol (Supplemental Fig. 24, 25).

## Discussion

We used multiple protocols and library preparations on HCT116 cells exposed to Nutlin-3a and determined that these experimental choices influence the signal of run-on sequencing libraries in systematic and often predictable ways. The shape of the characteristic gene initiation peak is strongly influenced by the underlying protocol, while the signal at gene elongation regions remain largely consistent across protocols. Likewise, the recovery of many intergenic regions was protocol specific, even when at high sequencing depths. Despite these differences, the ability to detect p53 activation was unaffected by the choice of enrichment or library preparation protocol.

Promoter proximal pausing is a pervasive feature of RNA polymerase II activity [15]. Pausing is often quantified through calculations of the pausing index, the ratio of reads within the initiation region relative to the elongation region. While PI values are known to depend on the choices of windows used to define these regions [15], our work demonstrates that they also depend on the underlying protocol even when the details of the PI index calculation are held constant. Furthermore, genes sometimes appear to have an additional pause site downstream of the annotated TSS (Fig. 3E) [42]. However, we have found that these second pause sites are protocol dependent; as changes in the library preparation method shift or ablate the signal of this second peak. While more work is necessary to fully characterize how protocol choices influence the precise location of the 5^′^ peak, it is clear that care must be taken when comparing 5^′^ distributions across experiments, as batch effects strongly influence this region.

Given the uniform activity of RNA polymerase II [43], the 5^′^ end protocol specific patterns we observed at genes should also impact enhancer associated transcripts. The most highly transcribed eRNAs (e.g. those annotated by FANTOM) are detected equally well by each protocol, but many eRNAs are lowly transcribed. Indeed, we observe that some enhancers with relatively high read coverage in one library are not detectable using a different protocol. We were surprised that increased depth did not resolve many of these protocol specific eRNAs. The variability in eRNA detection has likely hampered efforts to answer an outstanding question in the field; namely, how many eRNAs exist throughout the genome? Combining results from many different protocols and cell types may help alleviate this issue. This disparity in eRNA signal raises an intriguing question: which aspects of the protocols and resulting libraries contribute to the difference in eRNA capture rates? The slightly higher exon to intron ratio (Fig. 2D) of GRO-seq suggests this protocol contains a higher level of contaminating mRNA [44], consistent with Br-UTP antibody enrichment being a less efficient pull down method than Biotin-streptavidin enrichment. This bias also explains why GRO-seq has a higher gene to intergenic ratio compared to PRO-seq (Fig. 4A). These features may lead to some lowly transcribed eRNAs being more readily detectable with PRO-seq. In contrast, the use of Biotin halts polymerase elongation in PRO-seq, giving it a higher precision on RNA polymerase position [2]. However, this also results in short, unmappable fragments near the 5^′^ end of transcripts, which may limit the ability of PRO-seq to capture some shorter eRNAs. This phenomenon would explain why certain eRNAs are only captured in GRO-seq. Likewise, other factors probably contribute to the recovery of eRNAs [45], including sequence composition and biological variability.

Despite the observed protocol specific differences, our downstream analysis was consistent in detecting the underlying p53 perturbation. At genes, it is customary to exclude the initiation peak from differential gene transcription analysis [38–41], and our work indicates this is a wise choice, as counting reads only over elongation regions gave more consistent results across the protocols. Yet even when using only elongation regions, protocol specific batch effects determine which exact genes appear to respond, a problem also seen with RNA-seq [12, 46]. Likewise, detection of enhancer associated RNAs showed similar protocol specific batch effects. Importantly, despite the specifics of individual genes (and eRNAs) being not fully consistent, the large scale conclusion (p53 is activated by Nutlin-3a) remained consistent. Thus nascent transcription remains a powerful approach for understanding the immediate responses to perturbations including compounds and drug activity [5, 6, 40, 47].

## Conclusion

Protocol and platform differences have long been recognized as batch effect variables that introduce non-trivial experiment specific signals within high throughput sequencing data [48, 49]. Numerous efforts have focused on correcting batch effects, but it is always difficult to do so without some loss of biological signal [50, 51]. On the other hand, the distinct signals we detect raise an intriguing possibility that protocol and library preparation information can be inferred directly from the data itself. The noise component of the data can reliably differentiate between GRO- and PRO-seq datasets with remarkable accuracy, while sequence and quality signatures can often identify the library preparation methods used to prepare the dataset. Thus an automatic detection approach could be built to confirm or correct experimental information within the short read archive, at least for run-on assays [52]. Regardless, knowing the experimental details and managing associated batch effects is necessary when comparing in-house data to previously published data sets.

## Materials and methods

### Cell culture conditions

HCT116(ATCC cell line CCL-247, see [4]) and MCF10A(ATCC cell line CRL-10317 with a WTp53 insertion at p53 locus, see [53] for full information) cells were cultured in DMEM media supplemented with 10% FBS, 100 units/mL penicillin and 100 *μ*g/mL streptomycin, at 37^∘^C with 5% CO2. Cells were grown to a confluency of 60–70% in 15 cm culture dishes before passaging. Cells were passaged twice before harvesting, using PBS to wash and 0.05% w/v trypsin to detach the cells from the plate. Cells were aspirated and treated with media containing 10 *μ*M Nutlin-3a (or DMSO) for 1 hour before harvest.

### Nuclei isolation

Post-treatment, cells were placed on ice and washed three times with ice-cold PBS. Cells were incubated on ice in 10 mL ice-cold Lysis Buffer (10 mM Tris-HCl pH 7.5, 2 mM MgCl_2_, 3 mM CaCl_2_, 0.5% IGEPAL, 10% Glycerol, 2 U/mL SUPERase-IN, brought to volume with 0.1% DEPC DI-water, filtered before use) for 10 minutes. Cells were scraped and collected into 50 mL Falcon tubes, and centrifuged with a fixed-angle rotor at 1000 x g for 10 minutes at 4^∘^C. Cells were resuspended with Lysis buffer with a wide-opening P1000 tip, and washed twice with 10 mL Lysis buffer (centrifuged at 1000 x g for 5 minutes at 4^∘^C). After the second Lysis buffer wash, the samples were resuspended with 1 mL Freezing Buffer (50 mM Tris-HCl pH 8.3, 5 mM MgCl_2_, 40% Glycerol, 0.1 mM EDTA pH 8.0, brought to volume with 0.1% DEPC DI-water, filtered before use). Nuclei were centrifuged at 1000 x g for 5 minutes at 4^∘^C, and resuspended with 500 *μ*L Freezing Buffer. Nuclei were then centrifuged for 2 minutes at 2000 x g, 4^∘^C, and resuspended in 110 *μ*L Freezing Buffer. 10 *μ*L was retained for counting nuclei, while the remaining sample was snap-frozen in liquid nitrogen and stored at −80^∘^C until use.

### GRO-seq and library preparation methods

#### Ligation (LIG)

Run-on reactions were performed as in [1]. In brief, ice-cold isolated nuclei (100 *μ*L) were added to 37^∘^C 100 *μ*L reaction buffer (Final Concentration: 5 mM Tris-Cl pH 8.0, 2.5 mM MgCl_2_, 0.5 mM DTT, 150 mM KCl, 10 units of SUPERase In, 0.5% sarkosyl, 500 *μ*M rATP, rGTP, and Br-UTP, 2 *μ*M rCTP). The reaction was allowed to proceed for 5 min at 37^∘^C, followed by the addition of 23 *μ*L of 10X DNAseI buffer, and 10 *μ*L RNase free DNase I (Promega). RNA was extracted twice with Trizol, washed once with chloroform, and precipitated with 3 volumes of ice-cold ethanol and 1-2 *μ*L GlycoBlue. The pellet was washed in 75% ethanol before resuspending in 20 *μ*L of DEPC-treated water. Libraries were prepared as in [1]. In brief, nascent RNA was extracted and fragmented by base hydrolysis in 0.2 N NaOH on ice for 10–12 min, and neutralized by adding a 1 × volume of 1 M Tris-HCl pH 6.8. Fragmented nascent RNA was purified using Anti-BrdU beads and ligated with reverse 3 ^′^ RNA adaptor (/5Phos/GAUCGUCGGACUGUAGAACUCUGAAC/3InvdT/), and BrdU-labeled products were enriched by a second round of Anti-BrdU bead binding and extraction. For 5^′^ end repair, the RNA products were treated with tobacco acid pyrophosphatase (Epicenter) and T4 polynucleotide kinase (NEB). 5^′^ repaired RNA was ligated to reverse 5^′^ RNA adaptor (5^′^ UGGAAUUCUCGGGUGCCAAGG) before being purified by a final round of Anti-BrdU bead binding and extraction. RNA was reverse transcribed using 25 pmol of RP1 primer (5^′^AATGATACGGCGACCACCGAGATCTACACGTTCAGAGTTCTACAGTCCGA). The product was amplified 15 ±3 cycles and products >150 bp (insert > 70 bp) were size selected with 1X AMPure XP beads (Beckman) before being sequenced.

#### Random priming (RPR)

Run-on reactions were performed as in [1]. In brief, ice-cold isolated nuclei (100 *μ*L) were added to 37^∘^C 100 *μ*L reaction buffer (10mM Tris-Cl pH 8.0, 5 mM MgCl_2_, 1 mM DTT, 300 mM KCl, 20 units of SUPERase In, 1% sarkosyl, 500 *μ*M ATP, GTP, and Br-UTP, 2 *μ*M CTP). The reaction was allowed to proceed for 5 min at 30^∘^C, followed by the addition of 23 *μ*L of 10X DNAseI buffer, and 10 *μ*L RNase free DNase I (Promega). RNA was extracted twice with Trizol, washed once with chloroform, and precipitated with 3 volumes of ice-cold ethanol and 1-2 *μ*L GlycoBlue. The pellet was washed in 75% ethanol before resuspending in 20 *μ*L of DEPC-treated water. Libraries were prepared based on the NEBNext Ultra II Directional Library Preparation Kit. In brief, nascent RNA was extracted and fragmented by base hydrolysis in 0.2 N NaOH on ice for 10–12 min, and neutralized by adding a 1 × volume of 1 M Tris-HCl pH 6.8. Fragmented nascent RNA was purified using Anti-BrdU beads (Santa Cruz Biotech, Santa Cruz, CA) 3 times. Samples were reverse-transcribed using random hexamers, and sequencing adapters added by PCR. The product was amplified 15 ±3 cycles and products >150 bp (insert > 70 bp) were size selected with 1X AMPure XP beads (Beckman) before being sequenced.

### PRO-seq and library preparation methods

#### Ligation (LIG)

Run-on reactions were adapted from [3]. In brief, ice-cold isolated nuclei (100 *μ*L) were added to 37^∘^C 100 *μ*L reaction buffer (Final Concentration: 5 mM Tris-Cl pH 8.0, 2.5 mM MgCl_2_, 0.5 mM DTT, 150 mM KCl, 10 units of SUPERase In, 0.5% sarkosyl, 125 *μ*M rATP, 125 *μ*M rGTP, 125 *μ*M rUTP, 25 *μ*M biotin-11-CTP (additionally, two libraries generated with 25 *μ*M biotin-11-CTP, 250 *μ*M rCTP, see Supplemental Table 1). The reaction was allowed to proceed for 5 min at 37^∘^C. RNA was extracted twice with Trizol, washed once with chloroform, and precipitated with 3 volumes of ice-cold ethanol and 1-2 *μ*L GlycoBlue. The pellet was washed in 75% ethanol before resuspending in 20 *μ*L of DEPC-treated water. Nascent RNA was extracted and fragmented by base hydrolysis in 0.2 N NaOH on ice for 10–12 min, and neutralized by adding a 1 × volume of 1 M Tris-HCl pH 6.8. Fragmented nascent RNA was purified using streptavidin beads and ligated with reverse 3 ^′^ RNA adaptor (/5Phos/GAUCGUCGGACUGUAGAACUCUGAAC/3InvdT/), and biotin-labeled products were enriched by a second round of streptavidin bead binding and extraction. For 5^′^ end repair, the RNA products were treated with tobacco acid pyrophosphatase (Epicenter) and T4 polynucleotide kinase (NEB). 5^′^ repaired RNA was ligated to reverse 5^′^ RNA adaptor (5^′^ UGGAAUUCUCGGGUGCCAAGG) before being purified by a final round of streptavidin bead binding and extraction. RNA was reverse transcribed using 25 pmol of RP1 primer (5^′^AATGATACGGCGACCACCGAGATCTACACGTTCAGAGTTCTACAGTCCGA). The product was amplified 15 ±3 cycles and products >150 bp (insert > 70 bp) were size selected with 1X AMPure XP beads (Beckman) before being sequenced.

#### Template-switch reverse transcription (TSRT)

Template-Switch Reverse Transcription protocol (also known as uPRO), was adapted from [9]. Nuclei were incubated in the nuclear run-on reaction condition (5 mM Tris-HCl pH 8.0, 2.5 mM MgCl_2_, 0.5 mM DTT, 150 mM KCl, 0.5% Sarkosyl, 0.4 units / l of SUPERase-In) along with biotin-NTPs and rNTPs (125 *μ*M rATP, 125 *μ*M rGTP, 125 *μ*M rUTP, and 25 *μ*M biotin-11-CTP) for 5 min at 37^∘^C. Run-On RNA was extracted using TRIzol, and fragmented with 0.2 N NaOH for 10-12 min on ice. Fragmented RNA was neutralized with 1 M Tris-HCl pH 6.8, and buffer exchanged by passing through P-30 columns (Biorad). 3 ^′^ RNA adaptor (/5Phos/GAUCGUCGGACUGUAGAACUCUGAAC/3InvdT/) is ligated at 5 *μ*M concentration for 1 hour at room temperature using T4 RNA ligase (NEB), and nascent RNA was enriched twice with streptavidin beads. Extracted RNA was converted to cDNA using template switch reverse transcription with 1 *μ*M RP1-short RT primer (5^′^ GTTCAGAGTTCTACAGTCCGA), 3.75 M RTP-Template Switch Oligo (5^′^ GCCTTGGCACCCGAGAATTCCArGrGrG), 1x Template Switch Enzyme and Buffer (NEB) at 42^∘^C for 30 min. Resulting product was size selected with AMPure XP beads, and the cDNA was PCR amplified using primers compatible with Illumina Small RNA sequencing (TruSeq Small RNA primers RP1 and RPIn).

### Trimming, mapping, visualization, quality control

Resulting FASTQ files were trimmed and mapped to the GRCh38/hg38 reference genome and prepared for analysis and visualization through our in-house pipeline. In short, resulting FASTQ read files were first trimmed using bbduk (v38.05) to remove adapter sequences, as well as short or low quality reads. Reads were mapped with HISAT2 (v2.1.0), and resulting SAM files converted to BAM files using Samtools (v1.8). Reads with a mapping quality less than 5 were removed, which consequently also removed multi-mapping reads. BedGraph files were generated using Bedtools (v2.25.0), and converted to TDF files for visualization using IGVtools (v2.3.75). Quality metrics were generated with FastQC (v0.11.8), Preseq (v2.0.3), RSeQC (v3.0.0), with figures generated through MultiQC (v1.6). For further version information and specific input information, see NextFlow pipeline found at https://github.com/Dowell-Lab/Nascent-Flow.git.

### Exon/Intron ratio

RefSeq annotations were used to define exonic and intronic boundaries for each gene. The first exon of each gene was excluded (to avoid the initiation peak signal) in each calculation. To reduce the effect of noise, genes with low signal (RPKM < 1) were excluded from these calculations. Reads were counted using featureCounts from the R-Subread package (v1.6.0). Exonic and intronic reads were summed and normalized by RPKM, and a ratio for each gene is calculated. These ratios were log-normalized and the median ratio calculated for each set of libraries analyzed.

### Discrete wavelet transform

Samples with high coverage were used for this analysis. This included samples from the GRO-LIG, PRO-LIG, GRO-CIRC and PRO-TSRT libraries. The coverage over a gene transcript was normalized to 0-1 scale as show below:

$$c_{i} = \frac{x_{i} - min(x)}{max(x) - min(x)} $$

Where *x*=(*x*_*i*_,...,*x*_*n*_) represents read counts over a genomic location *n*, and *c*_*i*_ is the normalized coverage per genomic location. As we sought to identify protocol influences independent of biological gene variability, we limited our analysis to ubiquitously transcribed genes with low coefficient of variation (CV) across all samples. Thus, a total of 294 genes with a CV less than 0.55 and average transcripts per million (TPM) greater than 150 were selected. Using the PyWavelet (version 1.0.3) API in python (version 3.6.3), the symlet 5 mother wavelet was scanned across the 294 genes, returning wavelet coefficients (approximation coefficient and detail coefficients) (Fig. 2E) [20, 21, 54]. After the first pass of wavelet transform, the detail coefficients were used as input for principal component analysis (PCA) using scikit-learn (version 0.20.2) [55]. So, for each gene and each sample, PC1 and PC2 values were returned. Genes were split into categories based on whether the protocols could be split on PC1 and PC2 or whether the gene could not separate the protocols in PC space. The above process was then repeated for a larger set of 669 genes (CV less than 0.85 and average TPM greater than 100). Plots were generated with matplotlib (version 3.3.4), ggplot2 (version 3.3.3) and cowplot (version 1.1.1) [56–58]. Code for the DWT analysis can be found on github (https://github.com/Dowell-Lab/Protocol-Comparisons).

### Support vector machine

Principal component analysis values (from PC1 and PC2) derived from the wavelet transform analysis pipeline were used as input to a support vector machine (SVM). In order to verify the performance of the classification, the leave-one-out cross validation (LOOCV) criteria was used (Supplemental Fig. 6). A linear kernel was chosen for the SVM using the e1071 (version 1.7-4) package in R (R version 3.6.0) [59, 60]. The folds for the LOOCV were created with the caret package (version 6.0-86) in R (version 3.6.0) and accuracy for each fold and gene was calculated [61]. A total of 18 folds were created, where each of the 18 samples was held out one at a time as the test sample in the SVM, while the remaining samples were used as a training set. This was done for all the genes analysed and the evaluation determined the number of genes accurately predicting the protocol for each of the 18 samples. Plots were made using ggplot2 (version 3.3.3) and cowplot (version 1.1.1) [57, 58]. The jupyter notebook for the SVM LOOCV analysis can be found on github (https://github.com/Dowell-Lab/Protocol-Comparisons).

### Pause index calculations

Refseq annotations were used as the basis for pause index calculations. Counts were generated either from bedtools multicov (v2.28.0). The paused region was defined as -50 bp to 250 bp from the annotated TSS [23], and the elongation region was defined as 251 bp from the TSS to the annotated PolyA site. Reads from the same strand as the annotated gene were counted for the paused and elongation region, and calculated the index as follows:

$$\text{pausing\ index} (pi) = \frac{ReadCount(Pausing\ Region)/L1}{ReadCount\left(Gene\ Body\right)/L2} $$

Where L1 is the length of the pausing region (300 bp) and L2 is the length of the elongation region, measured from 251 bp past the TSS to the annotated cleavage site found in RefSeq. Only pause index values from a gene’s longest isoform were considered. Genes shorter than 2000 bp were removed.

The above analysis was repeated using featureCounts (v1.6.2) in the R-Subread package (v1.6.0), where the paused region was defined as -20 to +80 from the annotated TSS, and the elongation region as +81 from the TSS to -1000 from the annotated PolyA site. Genes shorter than 2000 bp were filtered out. These results are available in Supplemental Fig. 16.

### Simulation of reads near transcription start sites

We generated 2000 base gene template with equal proportions of A, C, G, and T. Using these templates, we then simulated RNA polymerase activity similar to a previously established mathematical framework [30]. Briefly, the model assumes a position for reads to start (the transcription start site) and a polymerase distribution around the TSS determined by a normal distribution. We sampled 10,000 initiation polymerases and 5,000 elongating polymerases randomly. Each polymerase was then allowed to run-on with a random change to terminate transcription based on the sequence identity and biotin-NTP/NTP ratio specified. Transcript lengths, e.g. reads, were then determined using the difference between the TSS and the termninated location of the polymerase. To mimic Ampure bead size selection, reads were then subjected to a size selection cutoff determined by an exponential distribution proportional to their length, resulting in an average cutoff of approximately 25 bases. The resulting read pool was subsequently used to generate metaplots of our synthetic template (Python v. 3.6.3, Numpy v.1.15.4, Pandas v. 0.23.4. Jupyter Notebook available at https://github.com/Dowell-Lab/Protocol-Comparisons).

### Short read ratio comparison

All reads greater than 30bp were filtered out of PRO-seq libraries to analyze the location of short reads within the genome. Each library was first assigned an Unlabeled/Labeled NTP ratio based on the run-on reaction concentrations of biotin-NTP relative to unlabeled NTPs reported by the authors for each dataset. GRO samples SRR14355674, SRR14355673, SRR14355662, SRR14355655 were included as a reference point. All PRO-seq libraries indicated in Supplemental Table 1 were considered for this analysis. Public samples SRR8033049, SRR8033050, SRR8033051, SRR8033052, SRR8033053, SRR8033054, SRR8033055, SRR8033056, SRR8033057, SRR8033058, SRR6205688, SRR6205689, SRR4041365, SRR4041366, SRR4041367, SRR4041368, SRR4041369, SRR4041370, SRR4041371, SRR4041372, SRR4041373, SRR5364303, and SRR5364304 were also included in this analysis, but were excluded from Supplemental Table 1 as they were not part of other analyses within this study.

Reads within 20 bp of the RefSeq TSS were considered to be near the TSS; we then calculated the ratio of these reads relative to all small reads found throughout the genome. The resulting ratio was plotted relative to the run-on reaction NTP ratio using R (version 3.6.3). Plots were made using ggplot2 (version 3.3.3) and cowplot (version 1.1.1) [57, 58].

### Gene/Intergenic reads ratio calculation

Genic and intergenic regions were determined by RefSeq (hg38, release number 109, downloaded August 14, 2019 from UCSC genome browser) annotation. Genic and intergenic read proportions were calculated by RSeQC (v3.0.0) read_distribution.py. Genic regions were defined as those overlapping a RefSeq annotation, including introns and untranslated regions. Intergenic regions were calculated as the remainder of reads not mapping to a gene region. The reads ratio of genic and intergenic regions can be found for each sample in Supplemental Table 1.

### Tfit

Tfit was used to identify regions of bidirectional transcription in each of our run-on sequencing libraries. Resultant BedGraph files from our samples were used as the input for the –bedgraph flag of the Tfit prelim module. The resultant preliminary region file was used as the –segment flag input for the Tfit model module, resulting in the final bidirectional calls used for analysis (see also https://github.com/Dowell-Lab/Tfit.git). Calls between replicates and treatments were combined using *muMerge*, generating a set of combined calls for each set of conditions (GRO-LIG, PRO-LIG, and GRO-CIRC). To compare library preparation methods, the above GRO-CIRC and GRO-LIG sets were combined together through bedtools merge (v2.28.0). Likewise, to compare enrichment methods, PRO-LIG and GRO-LIG sets were combined via bedtools merge (v2.28.0).

### dREG

We used dREG to identify regions of bidirectional transcription in each of our run-on sequencing libraries. Resultant BAM files from our samples were first converted to BigWig files compatible with dREG (see https://github.com/Danko-Lab/RunOnBamToBigWig.git). Using the online dREG portal, these files were used to generate dREG calls for bidirectional regions (https://django.dreg.scigap.org). Calls between replicates and treatments were combined using *muMerge*, generating a set of combined calls for each set of conditions (GRO-LIG, PRO-LIG, and GRO-CIRC). For comparative analyses between any of these sets, each set combined by *muMerge* was concatenated and used as the input for bedtools merge (v2.28.0), generating a consensus set of regions for those two sets.

### Differential transcription analysis

Differential transcription was performed using the DESeq2 (v1.26.0) R package (R version 3.6.3). DESeq2 no longer allows differential calls without replicates; thus, when comparing libraries where treatments and replicates were combined, the DESeq (v. 1.38.0) R package was used instead. Gene counts were generated using featureCounts (v1.6.2) from the R Subread package (v1.6.0), counting over the entire gene body from RefSeq Annotations (release number 109, downloaded August 14, 2019 from UCSC genome browser). For featureCounts, BED6 region files were converted to SAF format with the following command: awk -F "\t" -v OFS="\t" ’print{$4, $1, $2, $3, $6}’ region.bed > region.saf. Only the highest transcribed isoform of each gene was considered. Counts over Tfit, dREG, or FANTOM calls were generated with featureCounts.

### GSEA

DESeq2 gene results were ranked based on -log(*P*-value)/sign(Fold-Change). These ranked lists were used as the input for GSEA-preranked module (v4.1.0). The Hallmark v7.4 gene sets were used as the input database. Results were generated using 1000 permutations. Gene symbols were not collapsed.

### TFEA

Resulting Tfit bidirectional calls were used as the input for TFEA for each experiment (summarized in Supplemental Table 1). Calls were combined using *muMerge*. Transcription factor motifs were identified using FIMO (MEME Suite v5.1.1), using full human HOCOMOCO (version 11) motifs.

## Supplementary Information

**Additional file 1** **Supplemental Table 1** – Sample Information. Sample information for all RO-seq libraries used in analyses. Information is as follows: cell type, treatment, time point, enrichment protocol, library preparation method, replicate number, depth, complexity metrics, and SRA identifiers.

**Additional file 2** **Supplemental Figure 2** Metagenes of public GRO-RPR and in house libraries.
**Supplemental Figure 3** Read distribution of all libraries.
**Supplemental Figure 4** Discrete wavelet transform PCA results for 294 highly transcribed genes, demonstrates 39.8% of genes separate on PC1.
**Supplemental Figure 5** DWT PCA Results of detail coefficients at UBB Locus. PCA results for UBB locus, as in Figure 2F, but results are colored by library preparation method. At this locus, the results cluster less distinctly by library preparation method, compared to the enrichment protocol.
**Supplemental Figure 6** Schematic for the Support Vector Machine (SVM) leave one out cross validation (LOOCV) analysis. Eighteen nascent RNA sequencing samples were used as input. Given a gene, each of the samples was selected as a test sample and the other samples as training set, the SVM classification was evaluated. Based on this criteria, a majority of the genes (>75%) accurately classified the protocol for the n=18 samples.
**Supplemental Figure 7** Support vector machine results for 294 highly transcribed genes as well as a larger 669 set.
**Supplemental Figure 8** Scatterplot matrix of elongation region TPM for highly transcribed genes.
**Supplemental Figure 9** Heatmap showing the ratio of reads in pause regions between pairs of libraries.
**Supplemental Figure 11** Heatmap showing the ratio of reads in pause regions in publicly available data.
**Supplemental Figure 10** Metagenes for PRO-LIG libaries with varied biotin-NTP/NTP ratio.
**Supplemental Figure 12** Metagenes and pause index comparison in publicly available K562 data.
**Supplemental Figure 13** Short reads obtained from different run-on ratios and size selection criteria.
**Supplemental Figure 14** Ratio of small reads near TSS versus all short reads.
**Supplemental Figure 15** Scatterplot matrix of counts within the pause region of the most highly transcribed genes.
**Supplemental Figure 16** Pause index and rank correlation of GRO-CIRC and PRO-LIG libraries.
**Supplemental Figure 17** Comparison of signal in FANTOM enhancer annotations.
**Supplemental Figure 18** Upset plots of Tfit and dREG called regions across library preparations.
**Supplemental Figure 19** Example of an enhancer that is differentially recovered by different protocols.
**Supplemental Figure 20** Metagene summary of enhancers differentially detected between GRO-LIG and GRO-CIRC libraries.
**Supplemental Figure 21** GSEA enrichment plots for GRO-LIG, PRO-LIG and GRO-CIRC libraries.
**Supplemental Figure 22** Overlap of GSEA specified p53 genes in GRO-LIG and PRO-LIG libraries.
**Supplemental Figure 23** TFEA results for PRO-LIG libraries.
**Supplemental Figure 24** Example p53 responsive enhancer that is captured disparately across protocols.
**Supplemental Figure 25** Rank differential of GRO-LIG and PRO-LIG identified p53 responsive enhancers.

## Data Availability

The datasets used in this study are summarized in Supplemental Table 1. Datesets generated for this study are available through the Sequence Read Archive, under the accession PRJNA722106.

## Abbreviations

RO-seq: Run-On sequencing
PRO-seq: Precision Run-On sequencing
GRO-seq: Global Run-On sequencing
CIRC: Circularization based library preparation
LIG: Ligation based library preparation
RPR: Random Priming based library preparation
TSRT: Template Switching Reverse Transcriptase based library preparation
DWT: Discrete Wavelet Transform
PCA: Principal Component Analysis
SVM: Support Vector Machine
LOOCV: Leave-One-Out Cross Validation
TSS: Transcription Start Site
eRNA: Enhancer RNA
GSEA: Gene Set Enrichment Analysis
TFEA: Transcription Factor Enrichment Analysis
