# Supplementary material for: Protocol variations in run-on transcription dataset preparation produce detectable signatures in sequencing libraries
Source: BMC Genomics. 2022 Mar 7;23:187. doi: 10.1186/s12864-022-08352-8 (PMC8900324; doi:10.1186/s12864-022-08352-8)
Supplement: Supplementary file 2 — Additional file 2 Supplemental Figure 2 Metagenes of public GRO-RPR and in house libraries. Supplemental Figure 3 Read distribution of all libraries. Supplemental Figure 4 Discrete wavelet transform PCA results for 294 highly transcribed genes, demonstrates 39.8% of genes separate on PC1. Supplemental Figure 5 DWT PCA Results of detail coefficients at UBB Locus. PCA results for UBB locus, as in Figure 2F, but results are colored by library preparation method. At this locus, the results cluster less distinctly by library preparation method, compared to the enrichment protocol. Supplemental Figure 6 Schematic for the Support Vector Machine (SVM) leave one out cross validation (LOOCV) analysis. Eighteen nascent RNA sequencing samples were used as input. Given a gene, each of the samples was selected as a test sample and the other samples as training set, the SVM classification was evaluated. Based on this criteria, a majority of the genes (>75%) accurately classified the protocol for the n=18 samples. Supplemental Figure 7 Support vector machine results for 294 highly transcribed genes as well as a larger 669 set. Supplemental Figure 8 Scatterplot matrix of elongation region TPM for highly transcribed genes. Supplemental Figure 9 Heatmap showing the ratio of reads in pause regions between pairs of libraries. Supplemental Figure 11 Heatmap showing the ratio of reads in pause regions in publicly available data. Supplemental Figure 10 Metagenes for PRO-LIG libaries with varied biotin-NTP/NTP ratio. Supplemental Figure 12 Metagenes and pause index comparison in publicly available K562 data. Supplemental Figure 13 Short reads obtained from different run-on ratios and size selection criteria. Supplemental Figure 14 Ratio of small reads near TSS versus all short reads. Supplemental Figure 15 Scatterplot matrix of counts within the pause region of the most highly transcribed genes. Supplemental Figure 16 Pause index and rank correlation of GRO-CIRC and PRO-LIG libraries. Suppl [file 12864_2022_8352_MOESM2_ESM.pdf]

## Supplementary Material

# Protocol Variations in Run-on Transcription Dataset Preparation Produce Detectable Signatures in Sequencing Libraries

Samuel Hunter[1], Rutendo F. Sigauke[2],
Jacob T. Stanley[3], Mary A. Allen[1], Robin D. Dowell[1−4,*]

[1] BioFrontiers Institute, University of Colorado, Boulder CO 80309

[2] Computational Bioscience Program, Anschutz Medical Campus, University of Colorado, Aurora, CO 80045

[3] Department of Molecular, Cellular and Developmental Biology, University of Colorado, Boulder CO 80309

[4] Department of Computer Science, University of Colorado, Boulder CO 80309

∗ Corresponding author: robin.dowell@colorado.edu

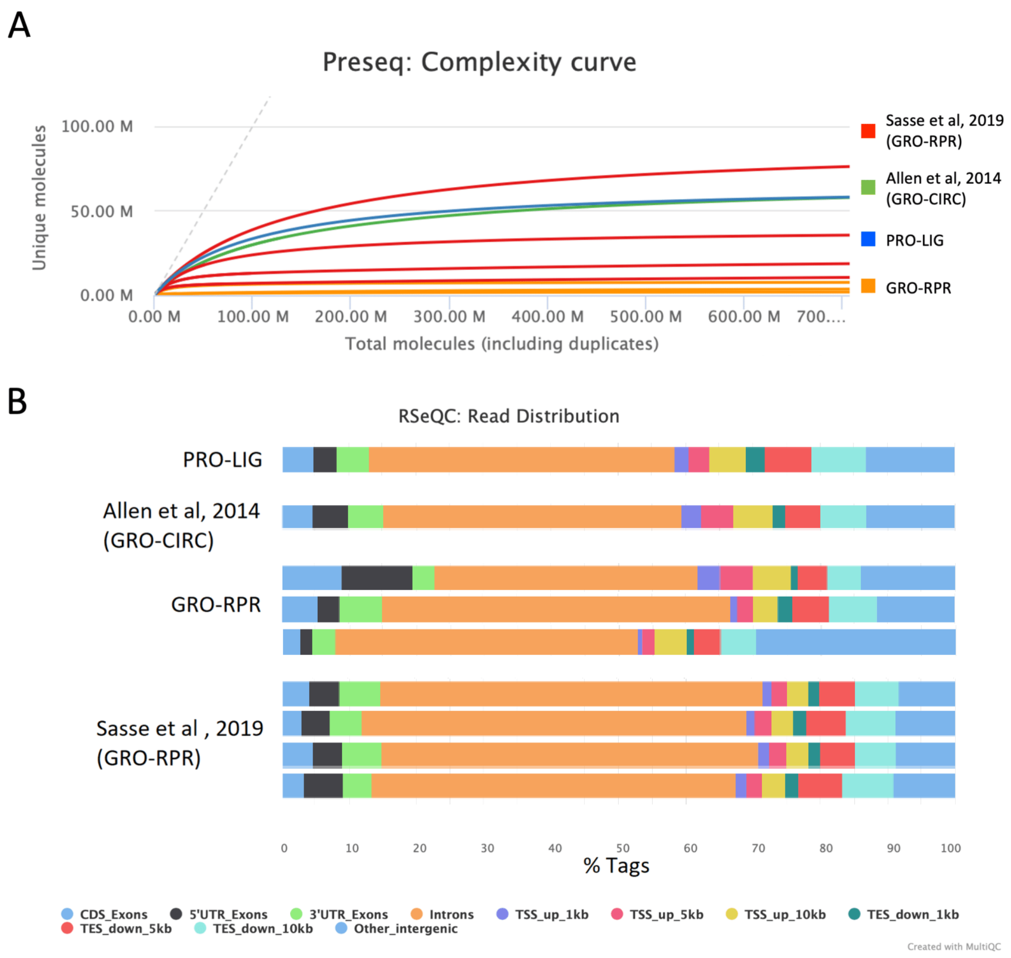


Supplementary Figure 1: **Preseq complexity curves and RSeQC Read Distribution Graphs of RPR Datasets** (A) Complexity curves of 4 publicly available GRO-RPR datasets ([5]: SRR8429046, SRR8429047, SRR8429054, SRR8429055), our in-house generated GRO-RPR datasets (see Supplemental Table 1, Materials and Methods. SRR14355654, SRR14355657, and SRR14355653), one PRO-LIG dataset (SRR14355672), and one publicly available GRO-CIRC dataset ([1]: SRR1105737). While the most complex library was from a GRO-RPR preparation, we found that the majority of these RPR datasets tended to be of lower complexity. Despite this trend, we contend that there is insufficient data to determine whether this is a fault of our handling or a feature of RPR library preparations with RO-seq datasets. (B) Read distribution plots of the datasets described in (A). While many regions were consistent regardless of protocol, was considerable variation in read distributions within the GRO-RPR datasets, especially comparing the proportion of reads found in 5' UTR regions and intergenic regions. As such, we chose to summarize additional quality metrics and library characteristics for our GRO-RPR datasets (Fig. 2D,3D,4B, see also Supplementary Table 1), with the understanding that their poor quality influence these metrics. GRO-RPR datasets were otherwise not used for further comparative analysis. From top to bottom, the samples are as follows: SRR14355672 (PRO-LIG); SRR1105737 (GRO-CIRC); SRR14355654, SRR14355657, SRR14355653 (GRO-RPR); SRR8429046, SRR8429047, SRR8429054, SRR8429055[5].

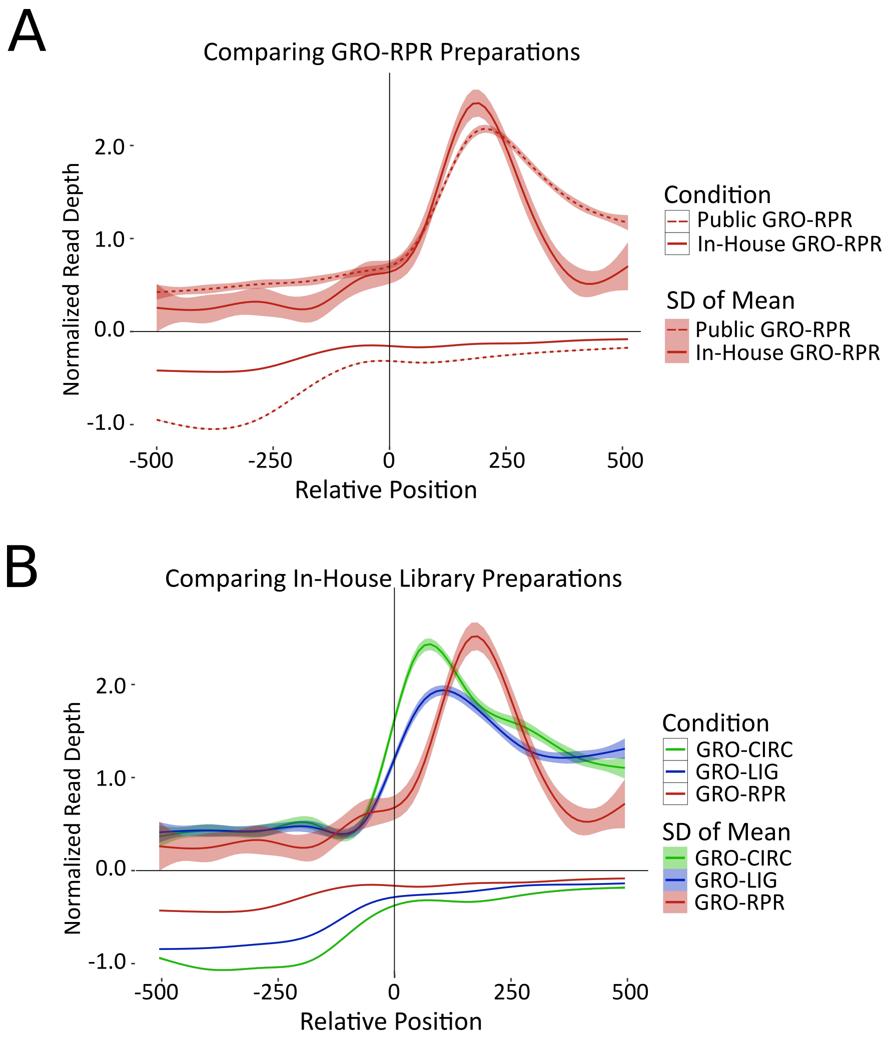


Supplementary Figure 2: **Metagenes of Public GRO-RPR and in-house libraries** (A) Metagenes of public GRO-RPR and in-house GRO-RPR libraries. All GRO-RPR datasets display a similar gap in coverage near the annotated TSS. Note that each public GRO-RPR library was subsampled to 20 million reads such that the comparison was performed at the same depth. (Public GRO-RPR data: SRR8429046, SRR8429047, SRR8429054, SRR8429055) (B) Metagenes of in-house libraries, including GRO-RPR libraries. Each library was subsampled to 20 million reads to match the lower depth of the GRO-RPR libraries. Additionally, we note that our GRO-RPR libraries are lower complexity. For both metaplots, genes shorter than 2000 bp, genes with significant signal 1 kb upstream (>1% of upstream bases covered), and genes with low coverage (TPM < .01) were removed. (n=1428) (GRO-CIRC: SRR1105736, SRR1105737. GRO-LIG: SRR14355673, SRR14355674. GRO-RPR: SRR14355653, SRR14355654, SRR14355657)

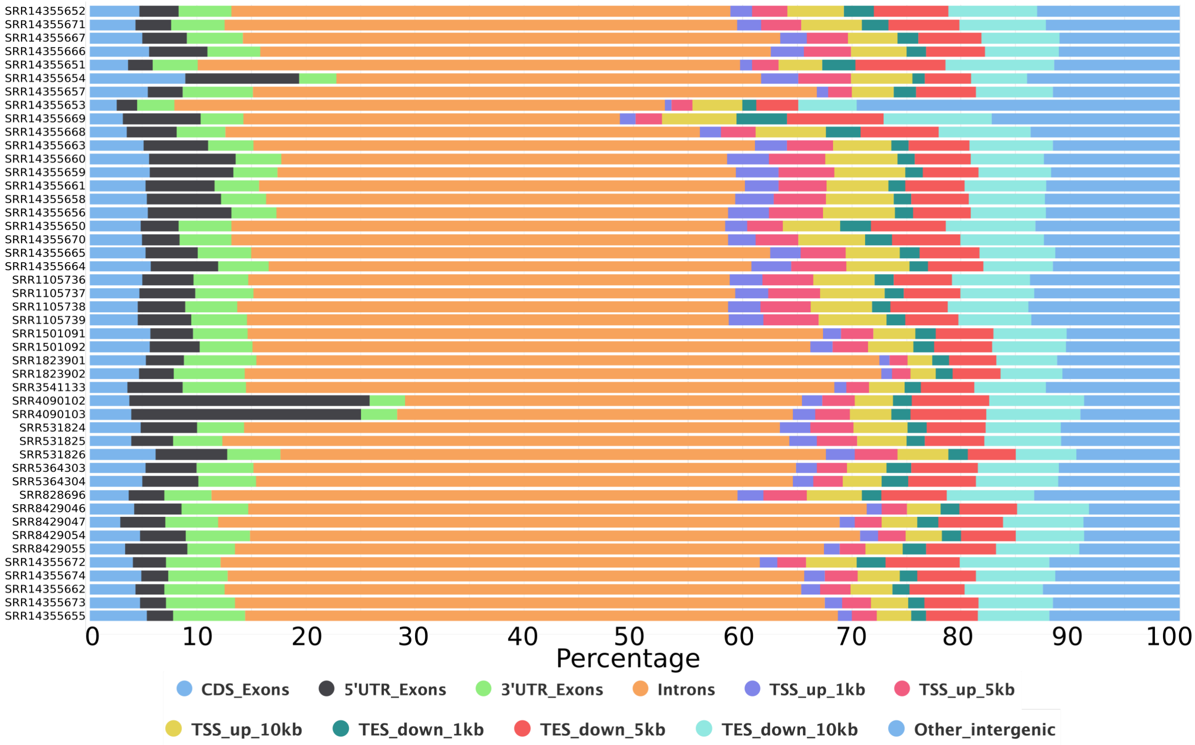


Supplementary Figure 3: **Read Distribution of all libraries in analysis** Read distributions were generated from RSeQC, see Materials and Methods, Supplemental Table 1.

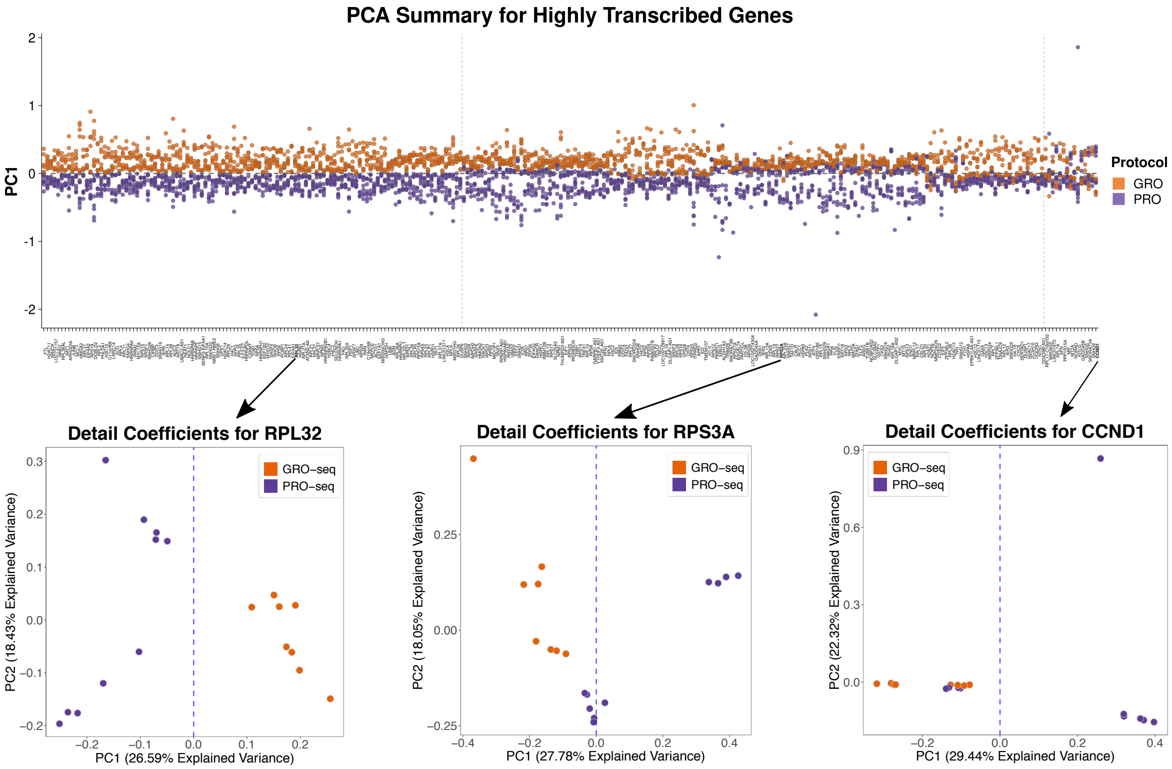


Supplementary Figure 4: **Discrete wavelet transform PCA results for 294 highly transcribed genes** (Top) PC1 effectively separates GRO and PRO libraries for 39.8% (117 genes) of the set of 294 highly transcribed genes while 55.1% (162 genes) of the genes separates the libraries on PC1 and PC2. (Bottom) PC1 and PC2 results for each library are shown for three example genes: RPL32 (separates on PC1), RPS3A (separates on a plane in the PC1/PC2 space), and CCND1 (not separable with these PC).

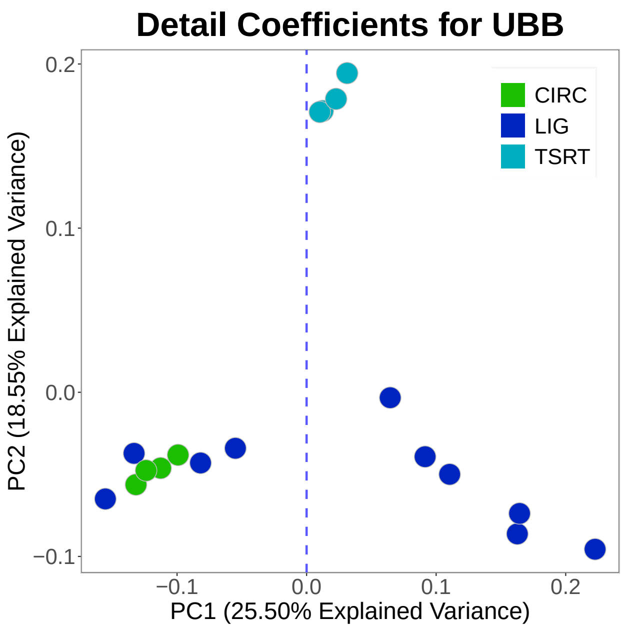


Supplementary Figure 5: **DWT PCA results of detail coefficients at UBB locus.** PCA results for UBB locus, as in Figure 2F. Results are colored by library preparation method. At this locus, the results cluster less distinctly by library preparation method, compared to the enrichment protocol.

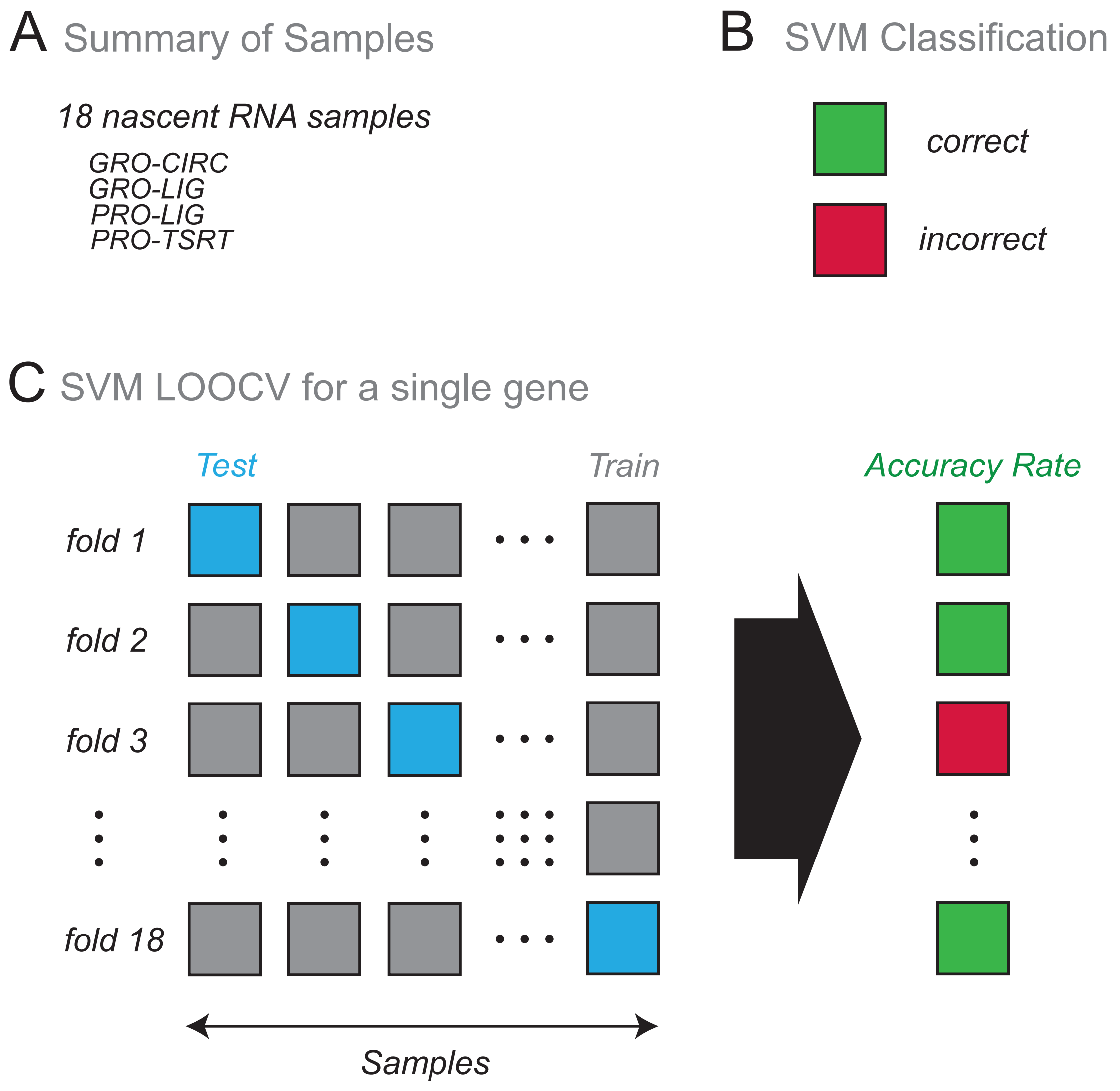


Supplementary Figure 6: **Schematic for the Support Vector Machine Leave one out cross validation analysis.** (A) Eighteen nascent RNA sequencing samples were used as input, from GRO-CIRC, GRO-LIG, PRO-LIG and PRO-TSRT libraries. (B) SVM classification was considered correct if the protocol was inferred from the data. (C) Given a gene, eighteen consecutive leave one out tests were performed. In each, one sample was selected as a test sample while the other samples were used as the training set. The SVM classification was subsequently evaluated for accuracy. Based on the SVM LOOCV method, a majority of the genes (>75%) accurately classified the protocol for the 18 samples.

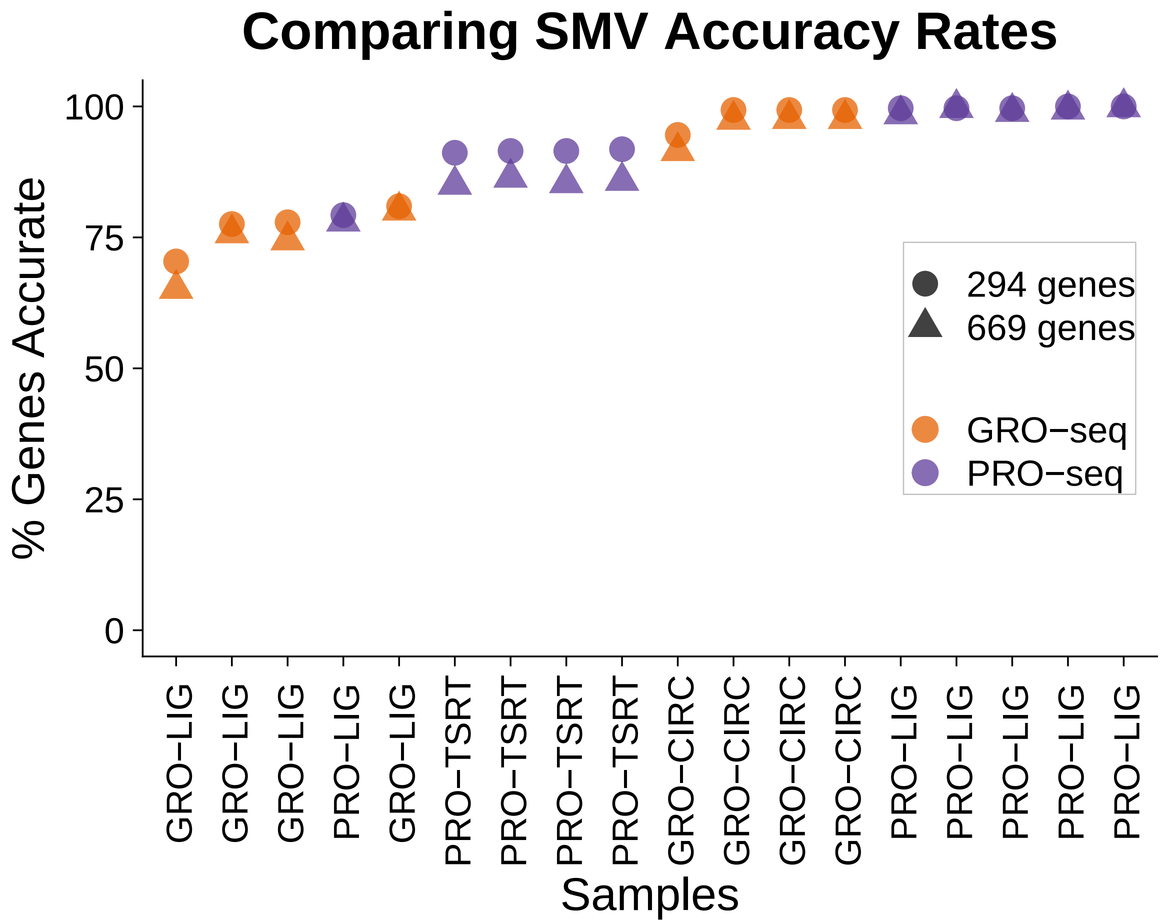


Supplementary Figure 7: **SVM results for highly transcribed genes.** The accuracy rate for the classifier remained mostly unchanged for both the top 294 and top 669 genes with high coefficient of variation (CV less than 0.85 and average TPM greater than 100).

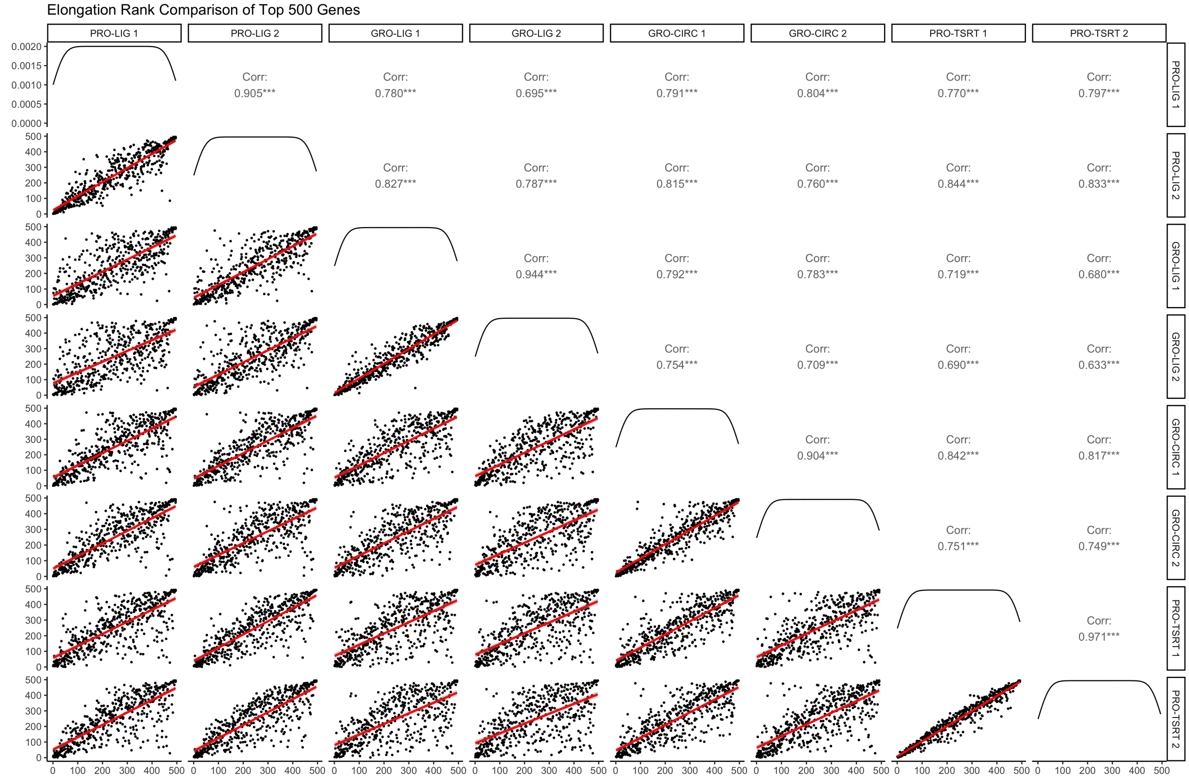


Supplementary Figure 8: **Scatterplot matrix of elongation regions.** Only the top 500 genes (by TPM) were considered. There is considerably more correlation in elongation regions versus pause regions at these genes, suggesting more variability occurs near the TSS across protocols. Each replicate dataset is a biological replicate (see Supplemental Table 1).

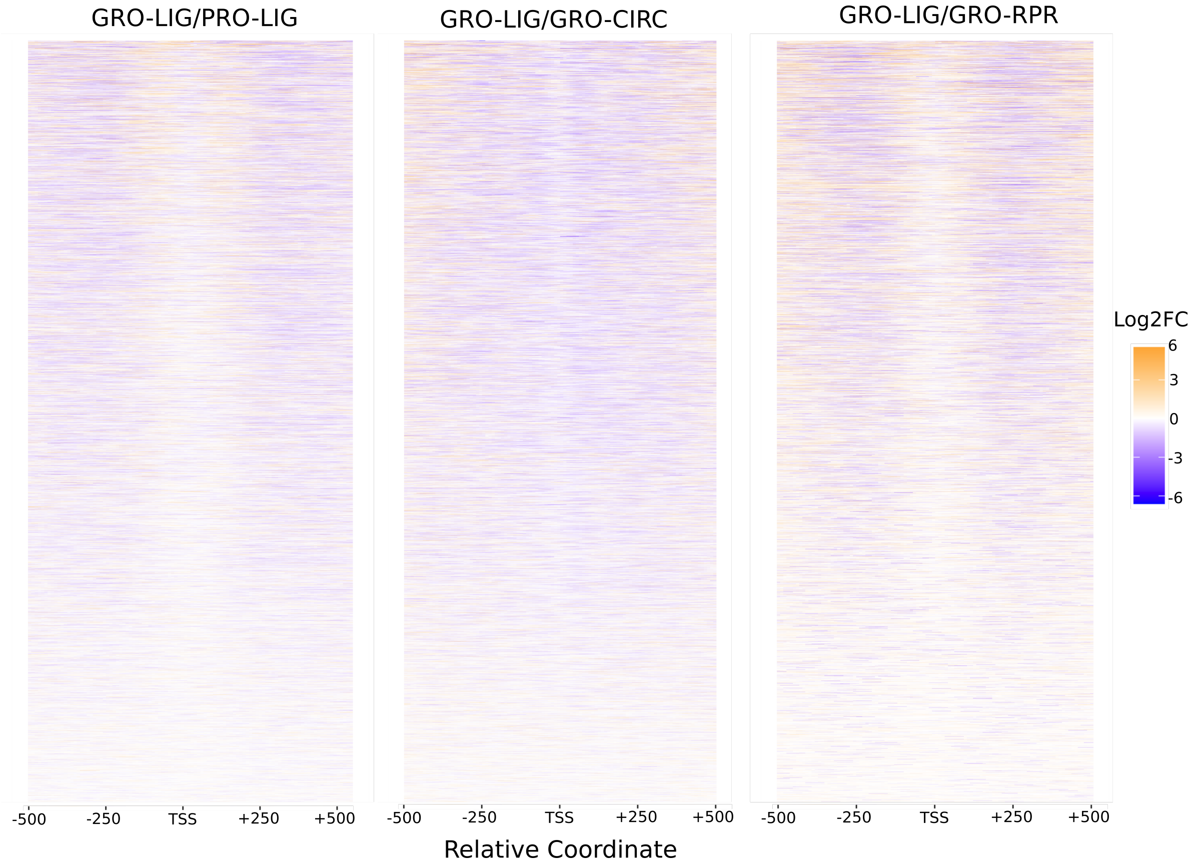


Supplementary Figure 9: **Heatmap of read ratios of pause regions in GRO-CIRC,GRO-LIG, GRO-RPR, and PRO-LIG libraries.** (TSS +/- 500 bp, 10 bp per window; RefSeq hg38 gene annotations were used.) Genes shorter than 2000 bp were not included. A pseudocount of 1 was added to all libraries to avoid undefined values. There is comparatively lower coverage near the TSS in many genes, representing the center of bidirectional transcription. This is especially prevalent in GRO-RPR and PRO-LIG libraries.

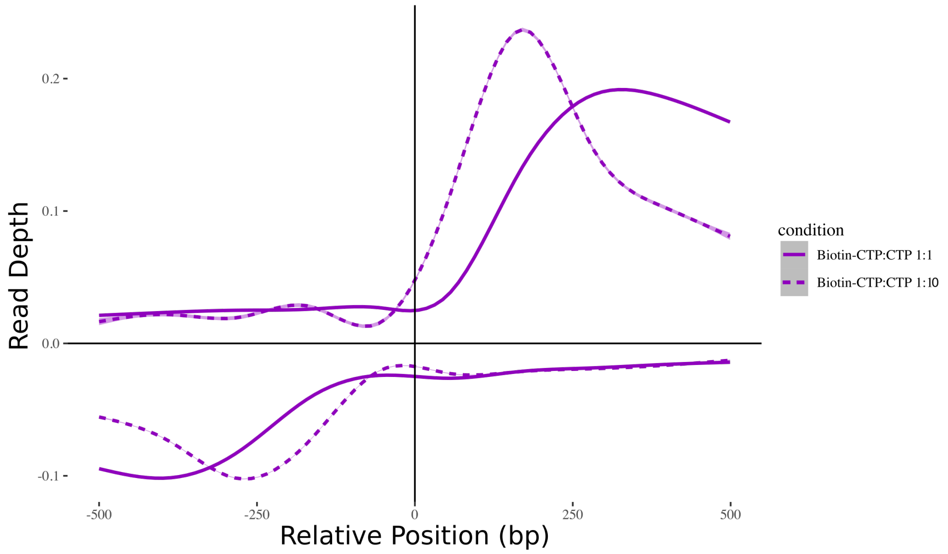


Supplementary Figure 10: **Metagenes of PRO-LIG libraries with varying Biotin ratios.** Libraries generated from HCT116 cell treated with DMSO, using the PRO-LIG protocol and library preparation strategies. Libraries differed only in the relative amounts of unlabeled CTP added (See Materials and Methods).

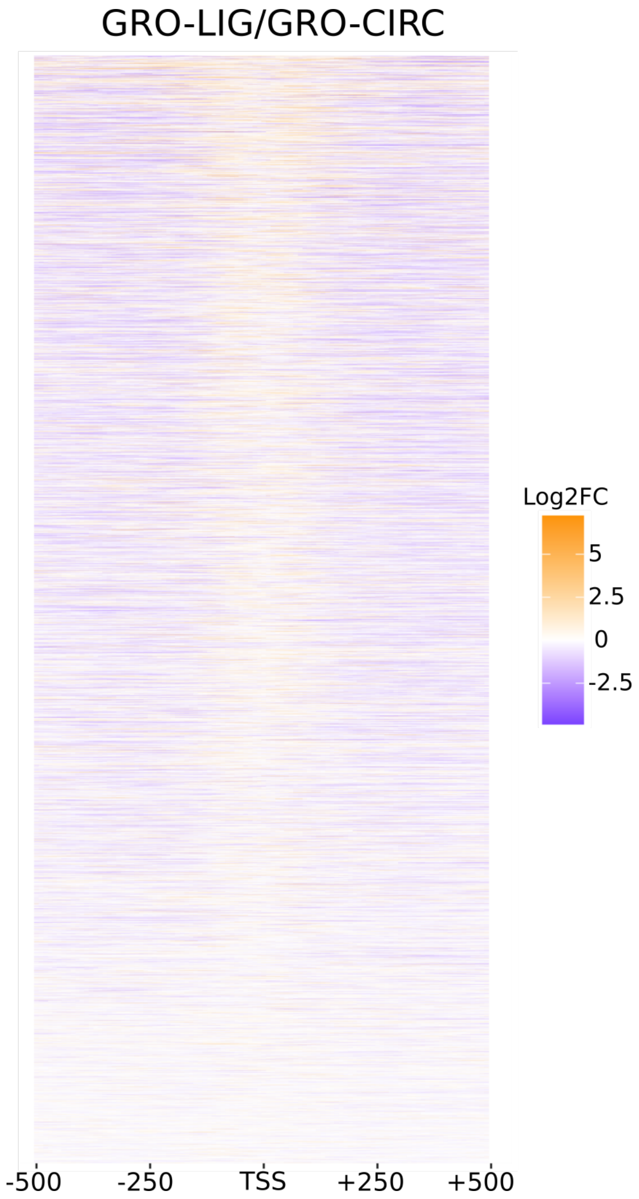


Supplementary Figure 11: **Ratio of reads near TSS in public datasets.** (TSS +/- 500 bp, 10 bp per window; RefSeq hg38 gene annotations were used.) Genes shorter than 2000 bp were not included. A pseudocount of 1 was added to all libraries to avoid undefined values. There is considerably more signal in the analyzed GRO-LIG library near the TSS, suggesting additional factors such as size selection contribute to disparities near these regions. (Public GRO-LIG: SRR1501091, SRR1501092; Public GRO-CIRC: SRR4090102, SRR4090103)

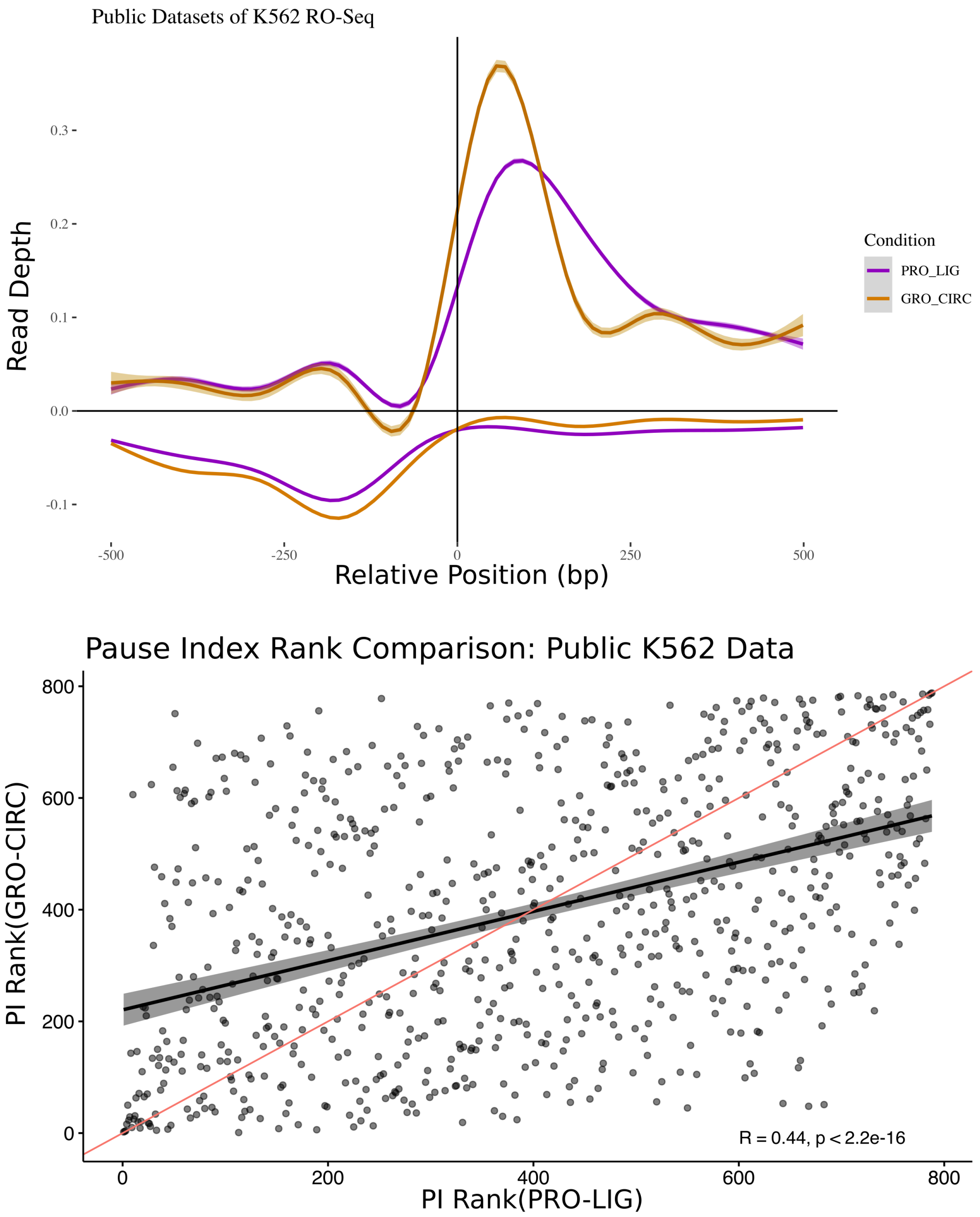


Supplementary Figure 12: **Metagene and Pause Index Comparison of Public K562 Data.** (Top) Metagenes of public datasets[4, 2]. Libraries were generated from K562 Cells treated with DMSO and prepped with either PRO-LIG or GRO-CIRC methods. PRO-LIG libraries were prepared with all 4 NTPs labeled with biotin during the run-on reaction. While the peak of these distributions occur at different relative locations than our datasets, we note that the PRO library still shows a peak that is further downstream than the comparative GRO library. (Bottom) Public data[4, 2] were subjected to analysis as in Fig. 3C, left (see Supplemental Table 1). PI regions were defined as in Fig. 3. Notably, the rank correlation remains low (R=0.44) consistent with PI differences being driven by protocol. Public GRO-CIRC: SRR1823901 and SRR1823902. Public PRO-LIG: SRR5364303 and SRR5364304, see Supplemental Table 1.

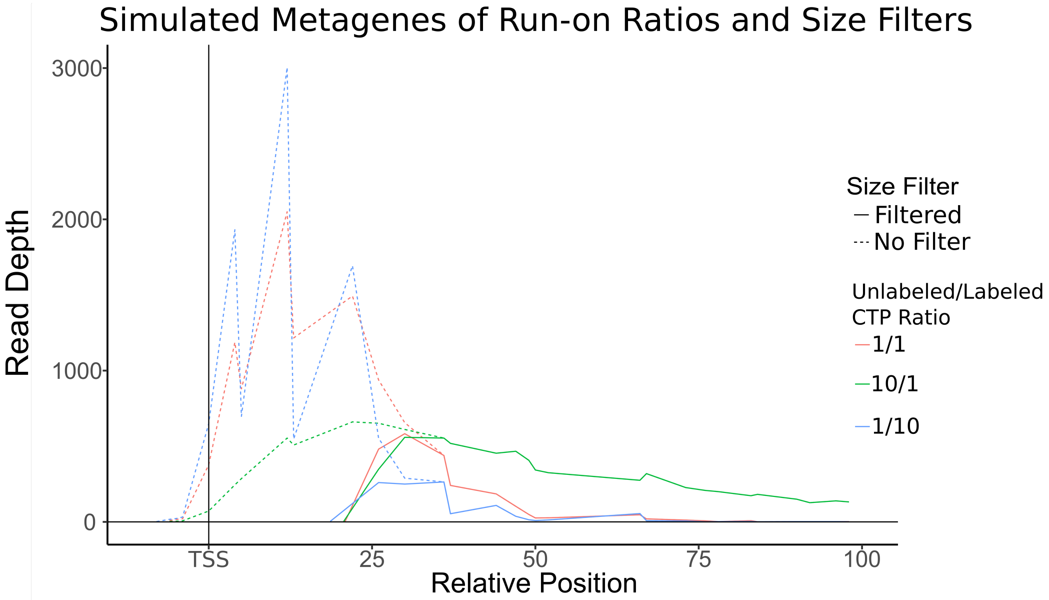


Supplementary Figure 13: **Simulated metagenes using different run-on ratios and size selection criteria.** Reads were generated *in silico* from a simulated gene template (see Materials and Methods), using run-on ratios to inform read positions and length. Small reads (approx. <25bp, see Materials and Methods) were either filtered out (solid lines), or kept in (dotted lines). As expected, with increasing NTP concentration the peak moves downstream (dashed lines). However, the size selection subsequently alters the location of the visible peak (solid lines) based on the proportion of the data that passes beyond the filter. In this way, the two protocol steps interact to influence the location observed for the 5′ peak. Here, for example, both the filtered 10/1 (green) and 1/1 (red) tracks report a 5′ peak near 28 bp, whereas the filtered 1/10 (blue) track reports a 5′ near 38 bp. Additionally, the read distribution in shifted towards the TSS in the filtered 1/1 track relative to the 10/1 track.

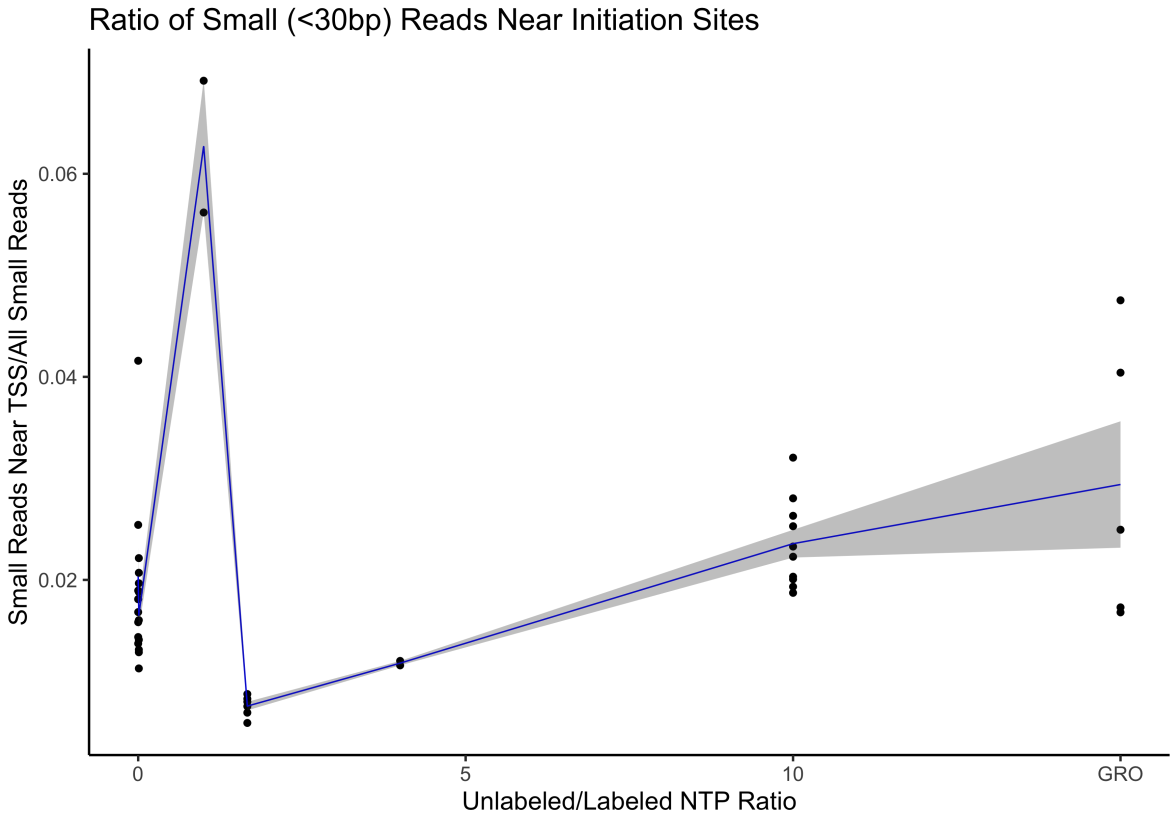


Supplementary Figure 14: **Ratio of small reads near TSS versus all small reads.** We reasoned that this ratio would be informative of the mixture of labeled and unlabeled NTPs in the run-on reaction. Based on publicly available data and our own in-house data (see Materials and Methods for full list of samples analyzed), there appears to be a trend in this ratio, although not a monotonically increasing function. The scarcity of different run-on ratios in public data do not warrant an estimate on an "ideal" ratio from these data; however, we note that these data are consistent with our in silico simulations.

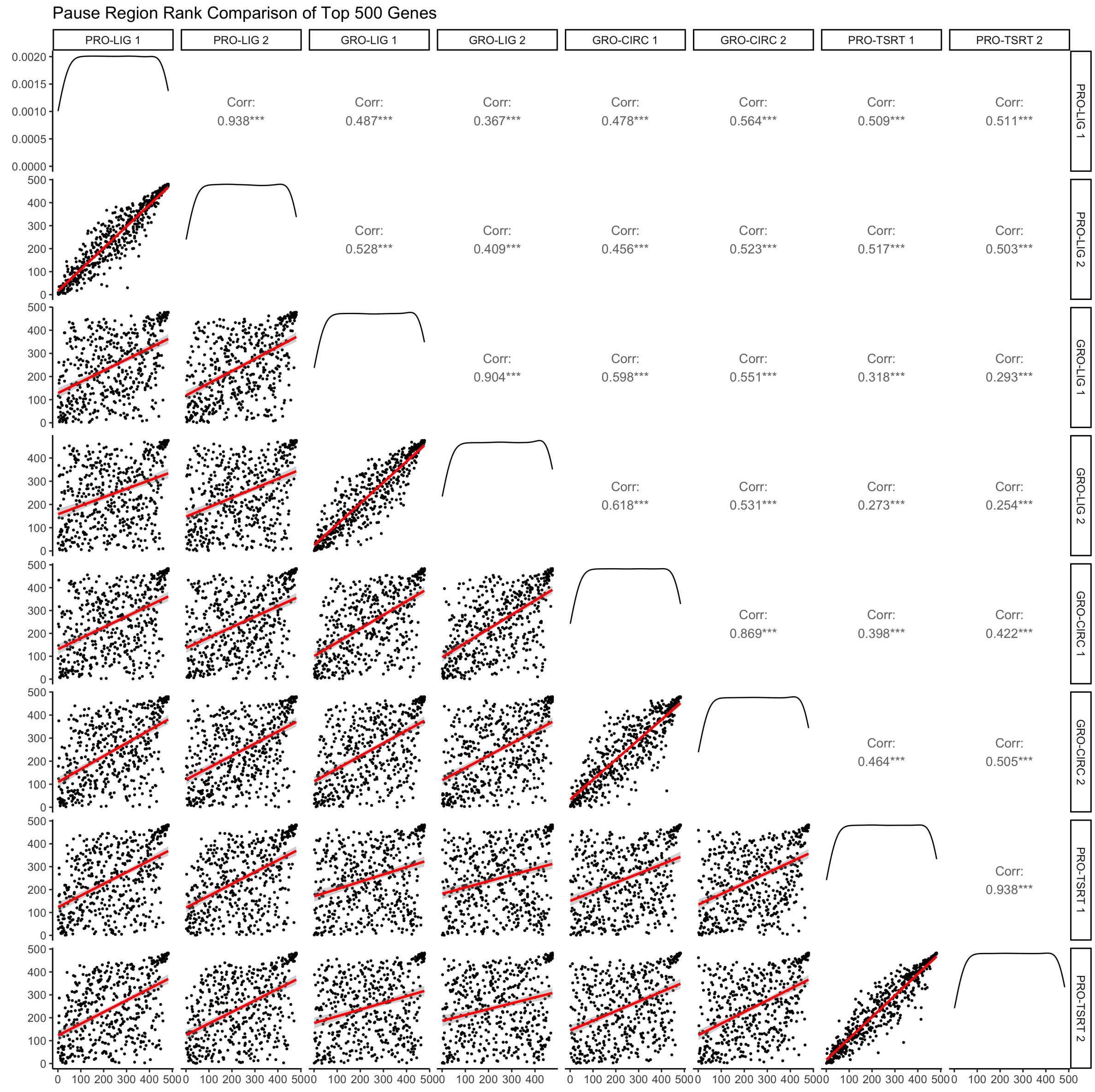


Supplementary Figure 15: **Scatterplot matrix of counts within the pause region of the top 500 genes.** (pause region:-50 to +250 from RefSeq hg38 TSS annotation). There is considerable variation between protocols at these regions. Replicates shown are biological replicates (see Supplemental Table 1).

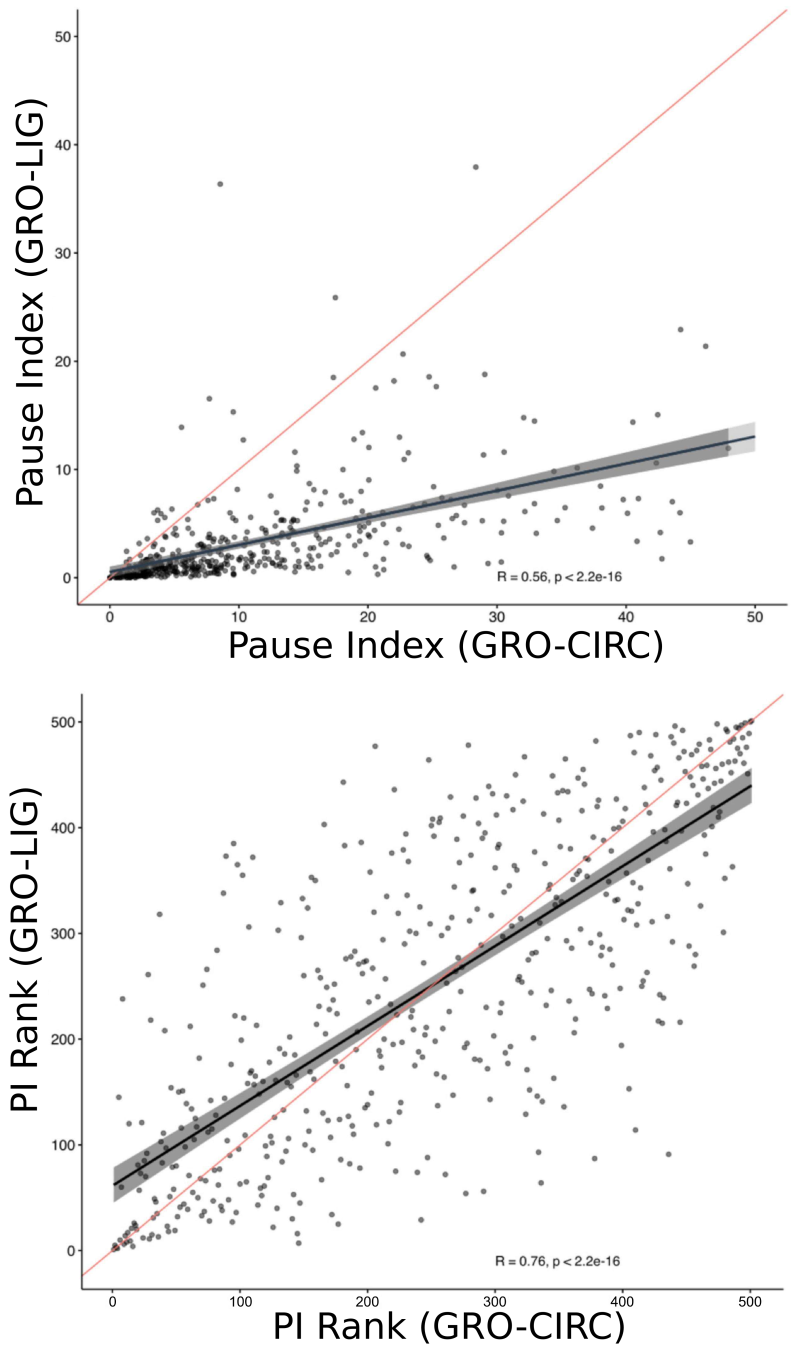


Supplementary Figure 16: **Pause index (PI) and rank correlation of PI generated from GRO-CIRC and GRO-LIG libraries.** Pause indices generated using a different pause region definition than Fig. 3E. Namely here the pause ratio is TSS to +80, elongation region +81:TES-1000 (genes shorter than 2000 bp were not included) and features were counted with featureCounts. In spite of using both a distinct interval and counting scheme, the pausing ratio remains poorly correlated (here Pearson R=0.56, Spearman R=0.76).

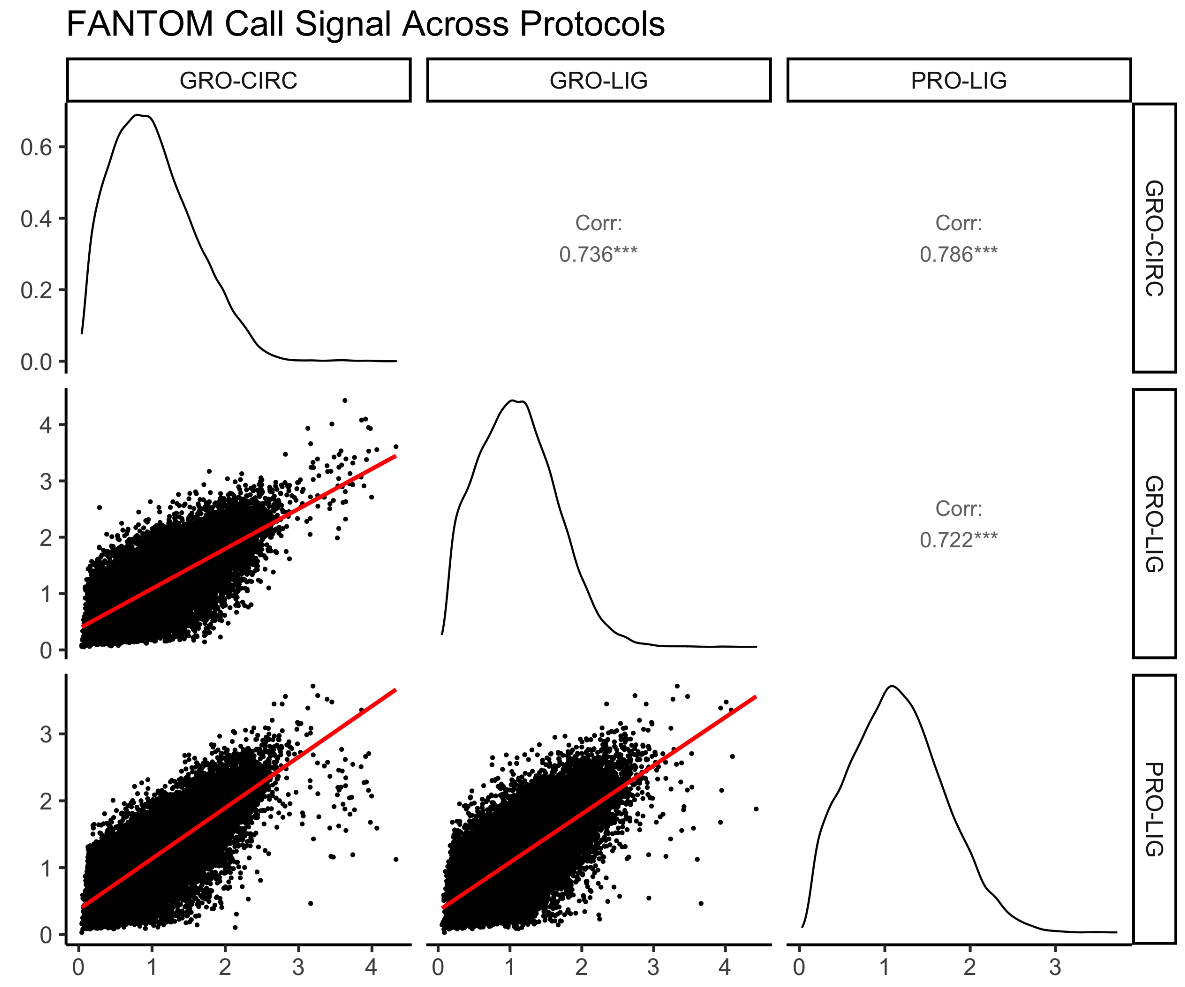


Supplementary Figure 17: **Scatterplot matrix of FANTOM regions.** FANTOM annotations [3] are generated from CAGE data, thus we reasoned that FANTOM annotated regions would be highly transcribed enhancers. Correlation levels are high between all protocols at these regions, albeit with considerable variation near select sites.

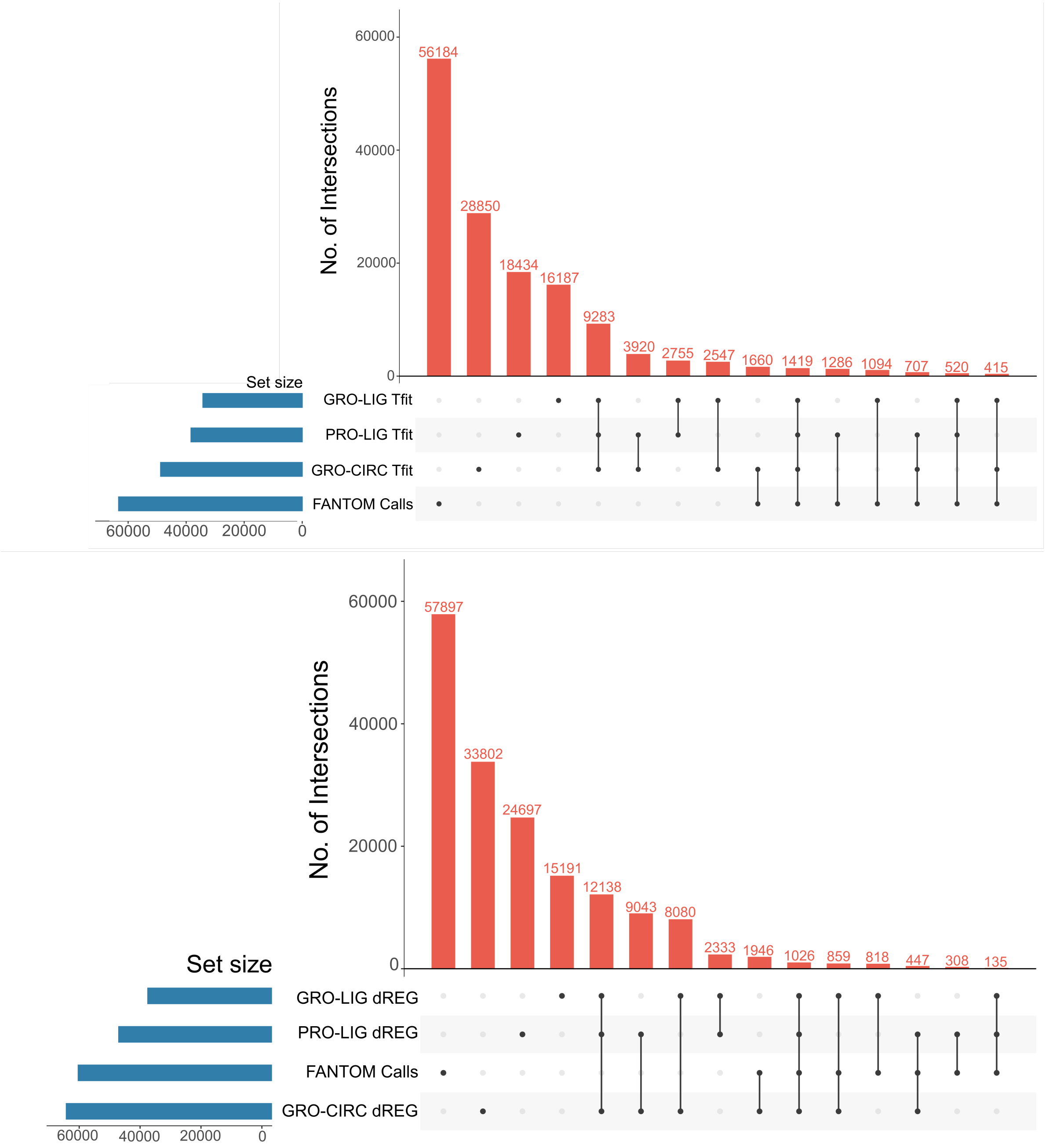


Supplementary Figure 18: **UpSet of Tfit/dREG calls among PRO-LIG, GRO-LIG, and GRO-CIRC libraries.** Bidirectional calls for equal numbers of DMSO-treated biological replicates were combined to form each set (PRO-LIG: n=2, combined depth 83.3 million reads (SRR14355652, SRR14355672); GRO-LIG: n=2, combined depth 108 million reads (SRR14355673, SRR14355674); GRO-CIRC: n=2, combined depth 212 million reads (SRR1105736, SRR1105737) (see Supplemental Table 1)). We observe frequent instances where each method does not call a region, despite the presence of bidirectional transcription, as shown in Fig. 4D,E. While this effect is depth dependent, there are notable regions where the strength of signal is strongly protocol dependent even after correcting for disparities in depth (Fig. 4G,H).

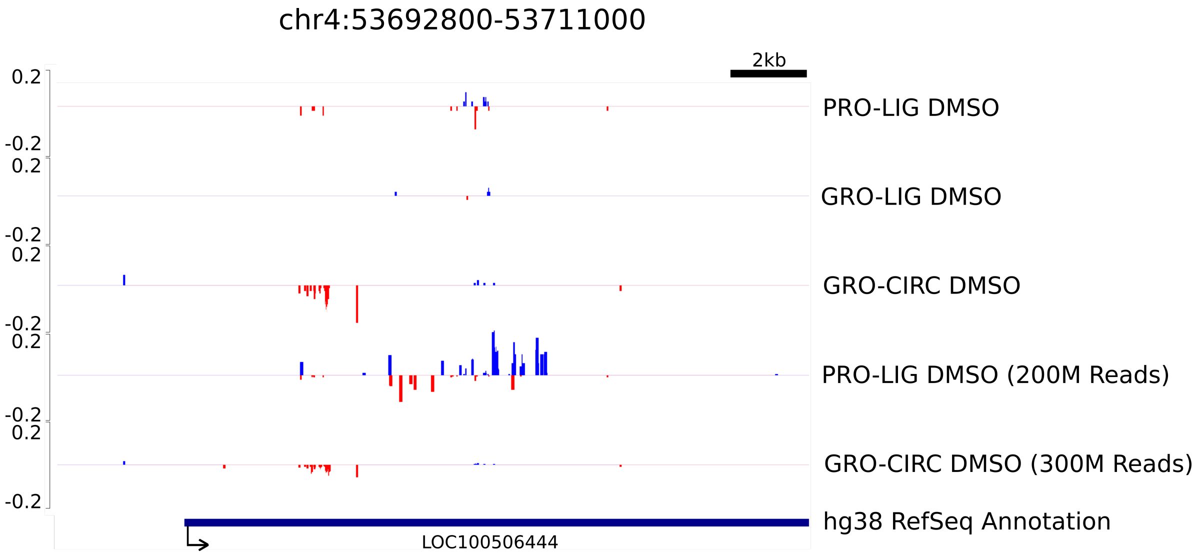


Supplementary Figure 19: **Example region indicating differences in enhancer transcription between protocols.** Read depths were normalized by CPM. Biological and technical replicates were combined to increase effective depth, as indicated in the bottom two read tracks (PRO-LIG: SRR14355650, SRR14355651 SRR14355652, SRR14355672; GRO-CIRC: SRR1105736, SRR1105737, SRR828696, see Supplemental Table 1).

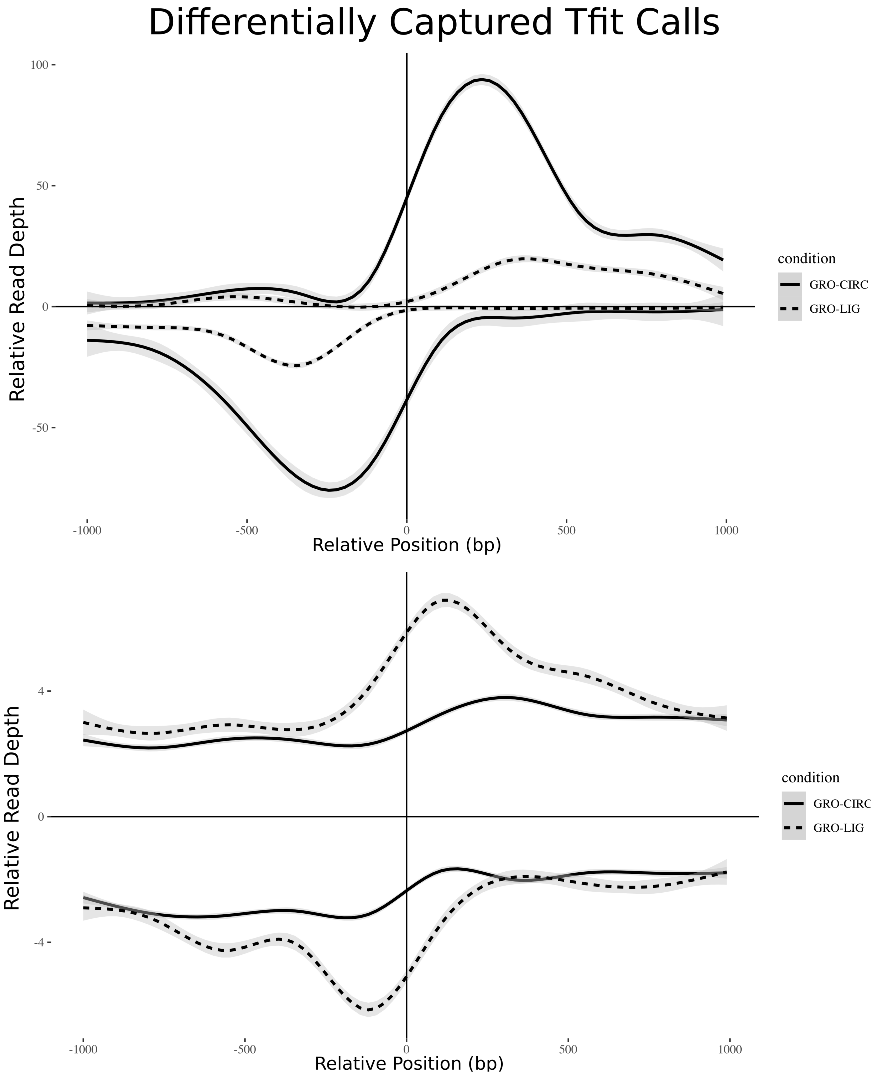


Supplementary Figure 20: **Metagene of enhancers differentially captured in either GRO-LIG or GRO-CIRC libraries.** Tfit calls across all replicates and treatments were combined together using *muMerge* for both GRO-LIG and GRO-CIRC libraries. Combined enhancers for GRO-LIG were then merged with combined enhancers for GRO-CIRC using bedtools merge (v2.28.0). Counts over these regions were used as input for DESeq1 (See also Materials and Methods). Differentially transcribed enhancers (Fig 4F, Materials and Methods) were used as inputs for metagene construction of GRO-CIRC (Top) and GRO-LIG (Bottom) preferentially obtained regions. Reads counts were normalized by CPM.

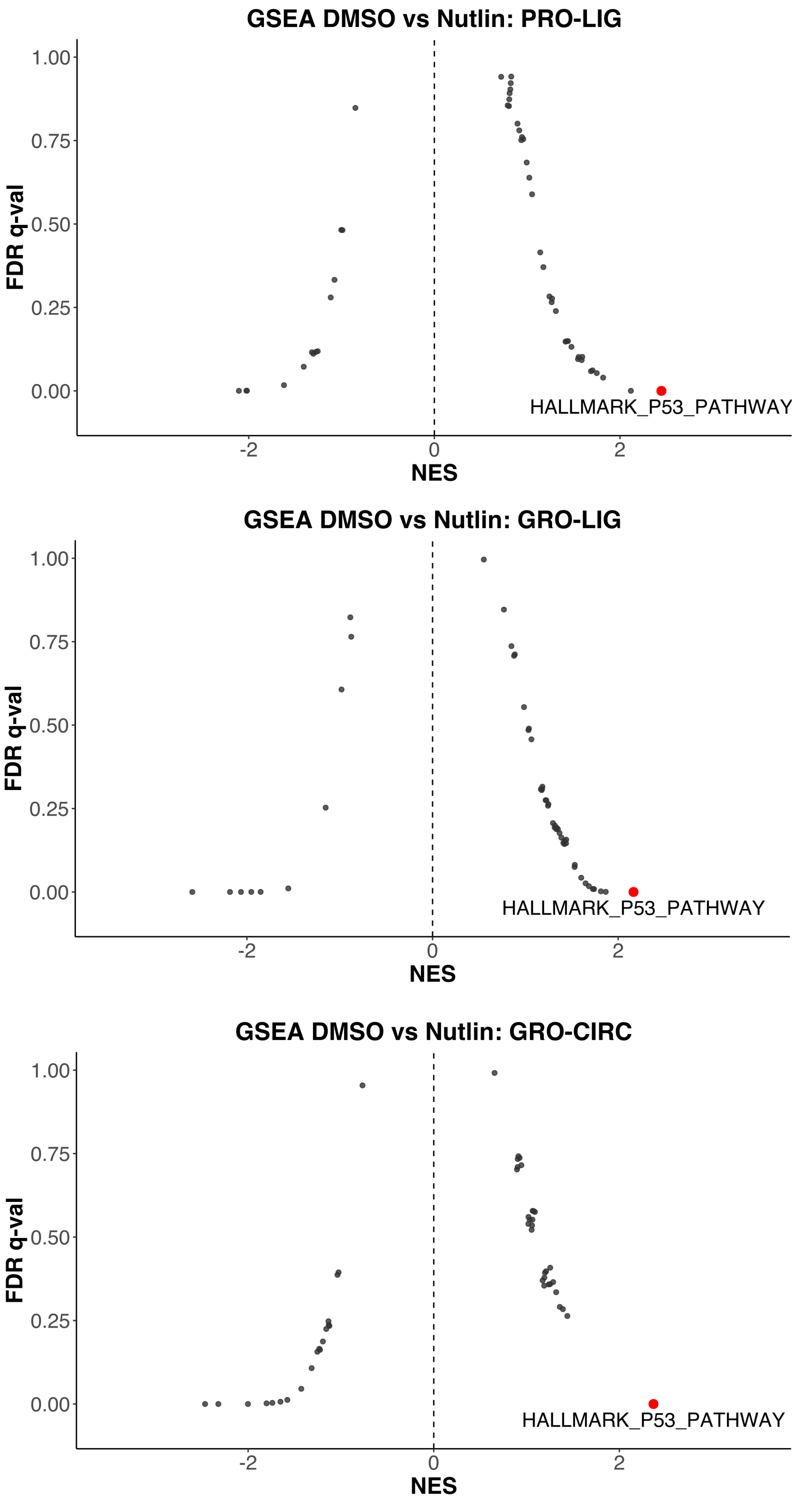


Supplementary Figure 21: **Enrichment plot of GSEA results for GRO-LIG, PRO-LIG, and GRO-CIRC libraries.** Gene region definitions were adjusted to exclude the 5′ pause peak, as per Fig 5A. In spite of library variations, the HALLMARK_P53_PATHWAY (red) is the strongest hit in all comparisons.

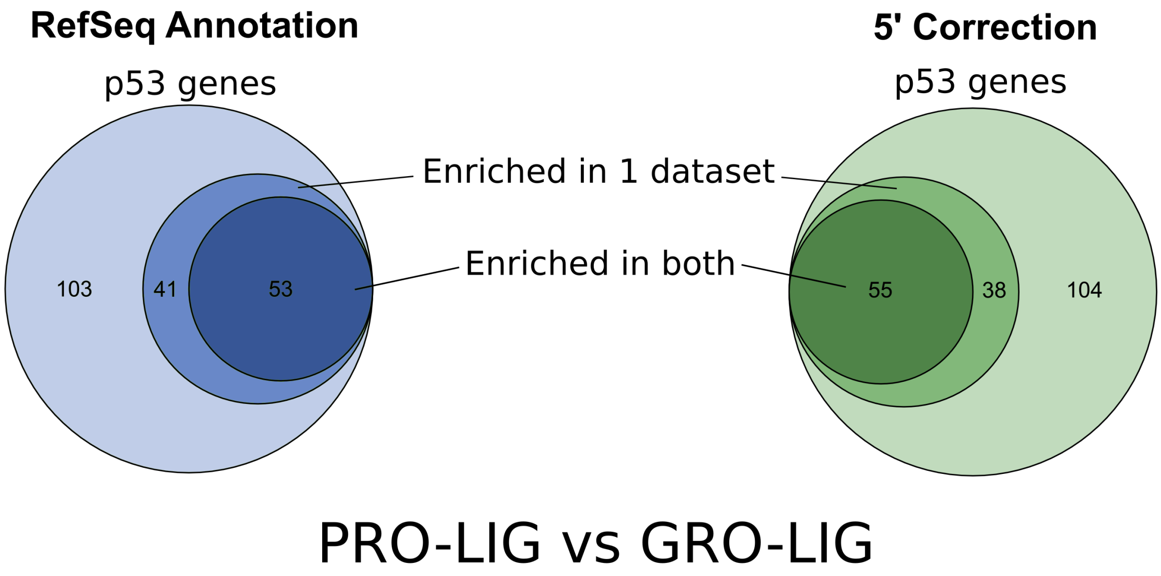


Supplementary Figure 22: **Overlap of GSEA p53 genes in GRO-LIG and PRO-LIG libraries.** Analysis was performed using counts over gene bodies (Left, hypergeometric test p-value=5.54e-15), and using a 5′ correction (Right, hypergeometric test p-value= 8.87e-17), as in Fig. 5A (see also Materials and Methods).

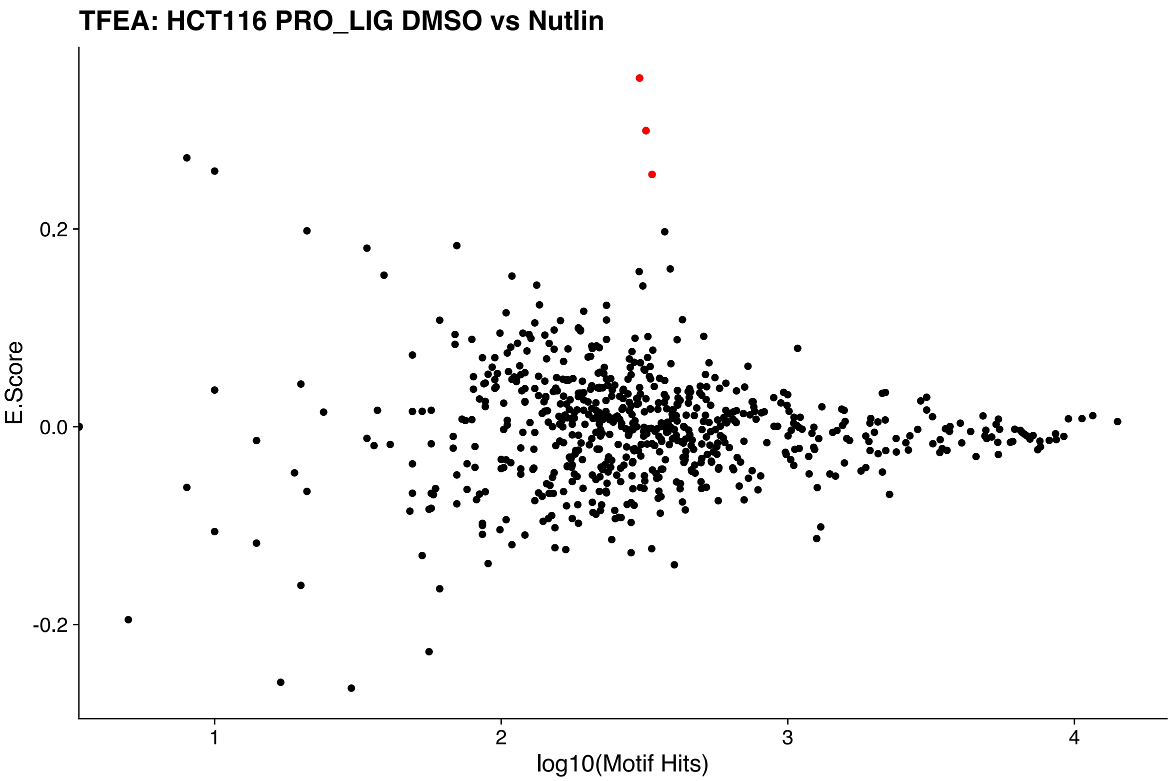


Supplementary Figure 23: **TFEA results for PRO-LIG libraries.** Regions were combined using *muMerge*, as in Fig. 5E,F. Red dots indicate transcription factors belonging to the p53 family (TP53, TP63, TP73).

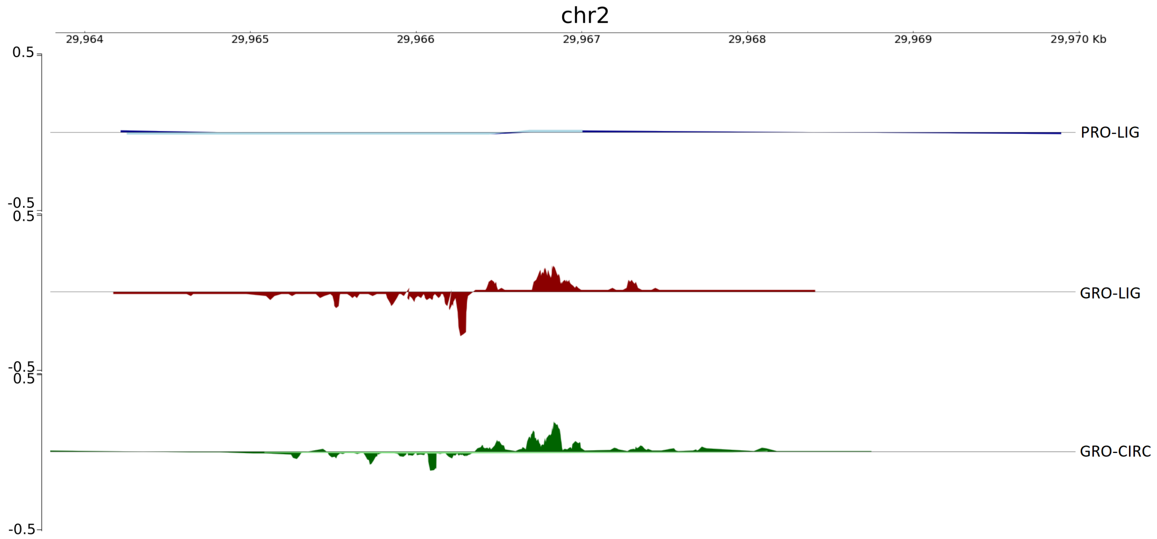


Supplementary Figure 24: **Example enhancer region where libraries disparately capture differential p53 enhancer activity.** Darker colors represent transcription level in Nutlin-3a treated libraries, while lighter colors represent levels found in DMSO-treated libraries. (Notably DMSO levels are nearly zero.) Read counts are normalized by CPM.

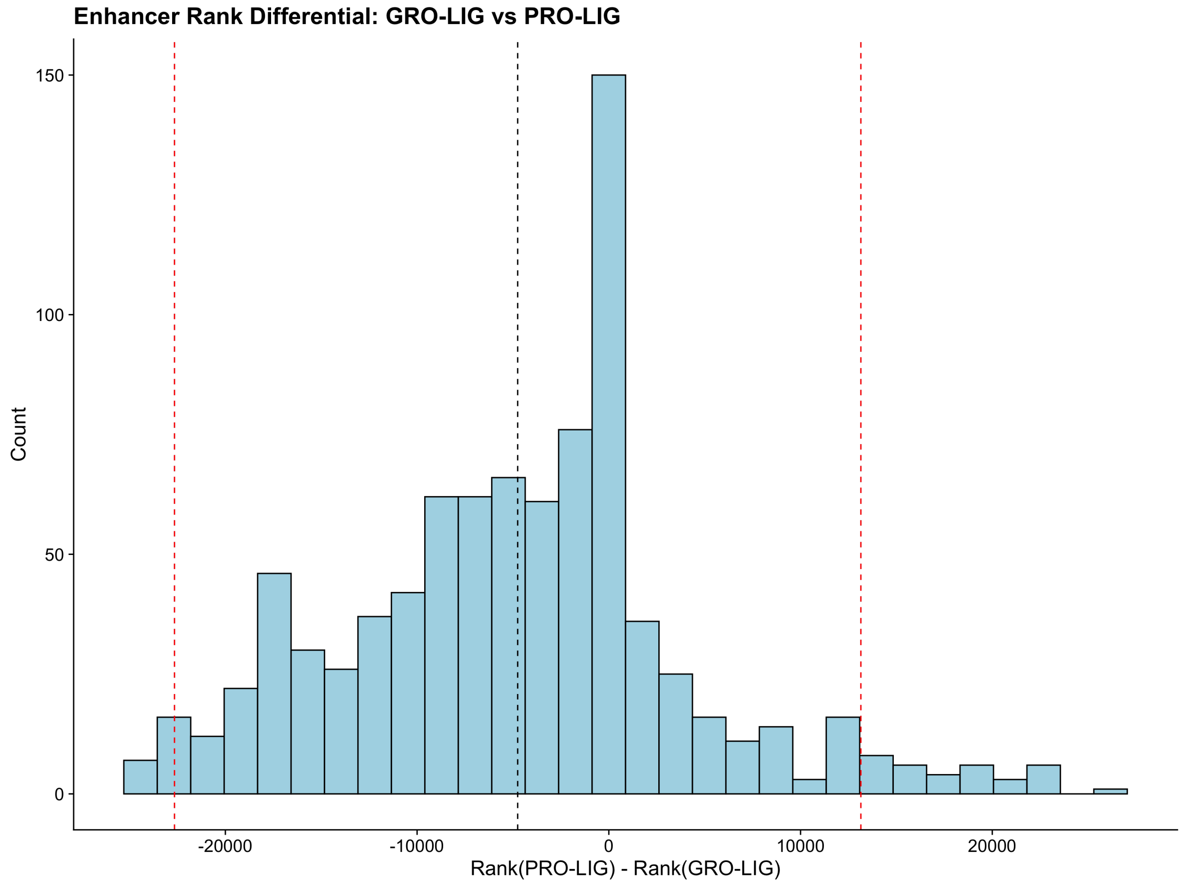


Supplementary Figure 25: **Rank differential of GRO-LIG and PRO-LIG enhancers.** Ranks were determined within TFEA through DESeq2. p53 enhancers which were more than 2 standard deviations (red dotted lines) from the mean (black dotted line) were considered to be differentially captured in GRO-seq or PRO-seq.

## Supplementary References

[1] M. A. Allen, H. Mellert, V. Dengler, Z. Andryzik, A. Guarnieri, J. A. Freeman, X. Luo, W. L. Kraus, R. D. Dowell, and J. M. Espinosa. Global analysis of p53-regulated transcription identifies its direct targets and unexpected regulatory mechanisms. *eLife*, 3:e02200, 2014. doi: 10.7554/eLife.02200.

[2] N. Dukler, G. T. Booth, Y.-F. Huang, N. Tippens, C. T. Waters, C. G. Danko, J. T. Lis, and A. Siepel. Nascent RNA sequencing reveals a dynamic global transcriptional response at genes and enhancers to the natural medicinal compound celastrol. *Genome Research*, 27(11):1816–1829, Oct. 2017.

[3] A. R. R. Forrest, H. Kawaji, M. Rehli, J. Kenneth Baillie, M. J. L. de Hoon, V. Haberle, T. Lassmann, I. V. Kulakovskiy, M. Lizio, M. Itoh, R. Andersson, C. J. Mungall, T. F. Meehan, S. Schmeier, N. Bertin, M. Jørgensen, E. Dimont, E. Arner, C. Schmidl, U. Schaefer, Y. A. Medvedeva, C. Plessy, M. Vitezic, J. Severin, C. A. Semple, Y. Ishizu, R. S. Young, M. Francescatto, I. Alam, D. Albanese, G. M. Altschuler, T. Arakawa, J. A. C. Archer, P. Arner, M. Babina, S. Rennie, P. J. Balwierz, A. G. Beckhouse, S. Pradhan-Bhatt, J. A. Blake, A. Blumenthal, B. Bodega, A. Bonetti, J. Briggs, F. Brombacher, A. Maxwell Burroughs, A. Califano, C. V. Cannistraci, D. Carbajo, Y. Chen, M. Chierici, Y. Ciani, H. C. Clevers, E. Dalla, C. A. Davis, M. Detmar, A. D. Diehl, T. Dohi, F. Drabløs, A. S. B. Edge, M. Edinger, K. Ekwall, M. Endoh, H. Enomoto, M. Fagiolini, L. Fairbairn, H. Fang, M. C. Farach-Carson, G. J. Faulkner, A. V. Favorov, M. E. Fisher, M. C. Frith, R. Fujita, S. Fukuda, C. Furlanello, M. Furuno, J.-i. Furusawa, T. B. Geijtenbeek, A. P. Gibson, T. Gingeras, D. Goldowitz, J. Gough, S. Guhl, R. Guler, S. Gustincich, T. J. Ha, M. Hamaguchi, M. Hara, M. Harbers, J. Harshbarger, A. Hasegawa, Y. Hasegawa, T. Hashimoto, M. Herlyn, K. J. Hitchens, S. J. Ho Sui, O. M. Hofmann, I. Hoof, F. Hori, L. Huminiecki, K. Iida, T. Ikawa, B. R. Jankovic, H. Jia, A. Joshi, G. Jurman, B. Kaczkowski, C. Kai, K. Kaida, A. Kaiho, K. Kajiyama, M. Kanamori-Katayama, A. S. Kasianov, T. Kasukawa, S. Katayama, S. Kato, S. Kawaguchi, H. Kawamoto, Y. I. Kawamura, T. Kawashima, J. S. Kempfle, T. J. Kenna, J. Kere, L. M. Khachigian, T. Kitamura, S. Peter Klinken, A. J. Knox, M. Kojima, S. Kojima, N. Kondo, H. Koseki, S. Koyasu, S. Krampitz, A. Kubosaki, A. T. Kwon, J. F. J. Laros, W. Lee, A. Lennartsson, K. Li, B. Lilje, L. Lipovich, A. Mackay-sim, R.-i. Manabe, J. C. Mar, B. Marchand, A. Mathelier, N. Mejhert, A. Meynert, Y. Mizuno, D. A. de Lima Morais, H. Morikawa, M. Morimoto, K. Moro, E. Motakis, H. Motohashi, C. L. Mummery, M. Murata, S. Nagao-Sato, Y. Nakachi, F. Nakahara, T. Nakamura, Y. Nakamura, K. Nakazato, E. van Nimwegen, N. Ninomiya, H. Nishiyori, S. Noma, T. Nozaki, S. Ogishima, N. Ohkura, H. Ohmiya, H. Ohno, M. Ohshima, M. Okada-Hatakeyama, Y. Okazaki, V. Orlando, D. A. Ovchinnikov, A. Pain, R. Passier, M. Patrikakis, H. Persson, S. Piazza, J. G. D. Prendergast, O. J. L. Rackham, J. A. Ramilowski, M. Rashid, T. Ravasi, P. Rizzu, M. Roncador, S. Roy, M. B. Rye, E. Saijyo, A. Sajantila, A. Saka, S. Sakaguchi, M. Sakai, H. Sato, H. Satoh, S. Savvi, A. Saxena, C. Schneider, E. A. Schultes, G. G. Schulze-Tanzil, A. Schwegmann, T. Sengstag, G. Sheng, H. Shimoji, Y. Shimoni, J. W. Shin, C. Simon, D. Sugiyama, T. Sugiyama, M. Suzuki, N. Suzuki, R. K. Swoboda, P. A. C. 't Hoen, M. Tagami, N. Takahashi, J. Takai, H. Tanaka, H. Tatsukawa, Z. Tatum, M. Thompson, H. Toyoda, T. Toyoda, E. Valen, M. van de Wetering, L. M. van den Berg, R. Verardo, D. Vijayan, I. E. Vorontsov, W. W. Wasserman, S. Watanabe, C. A. Wells, L. N. Winteringham, E. Wolvetang, E. J. Wood, Y. Yamaguchi, M. Yamamoto, M. Yoneda, Y. Yonekura, S. Yoshida, S. E. Zabierowski, P. G. Zhang, X. Zhao, S. Zucchelli, K. M. Summers, H. Suzuki, C. O. Daub, J. Kawai, P. Heutink, W. Hide, T. C. Freeman, B. Lenhard, V. B. Bajic, M. S. Taylor, V. J. Makeev, A. Sandelin, D. A. Hume, P. Carninci, Y. Hayashizaki, T. F. Consortium, the RIKEN PMI, and C. (DGT). A promoter-level mammalian expression atlas. *Nature*, 507(7493):462–470, 2014.

[4] E. A. Niskanen, M. Malinen, P. Sutinen, S. Toropainen, V. Paakinaho, A. Vihervaara, J. Joutsen, M. U. Kaikkonen, L. Sistonen, and J. J. Palvimo. Global sumoylation on active chromatin is an acute heat stress response restricting transcription. *Genome Biology*, 16(1):153, Jul 2015.

[5] S. K. Sasse, M. Gruca, M. A. Allen, V. Kadiyala, T. Song, F. Gally, A. Gupta, M. A. Pufall, R. D. Dowell, and A. N. Gerber. Nascent transcript analysis of glucocorticoid crosstalk with TNF defines primary and cooperative inflammatory repression. *Genome Research*, 2019.
